# Robot-assisted gait training in people with subacute and chronic stroke: a randomized controlled trial

**DOI:** 10.3389/fneur.2026.1898475

**Published:** 2026-09-03

**Authors:** Michaela Stampfer-Kountchev, Sarah Mildner, Isabella Hotz, Janice Habig, Markus Pröglhöf, Johanna Panzl, Christian Brenneis, Barbara Seebacher

**Affiliations:** 1 Department of Neurology, Clinic for Rehabilitation Münster, Münster, Austria; 2 Department of Rehabilitation Science, Clinic for Rehabilitation Münster, Münster, Austria; 3 Karl Landsteiner Institute for Interdisciplinary Rehabilitation Research, Münster, Austria; 4 Clinical Department of Neurology, Medical University of Innsbruck, Innsbruck, Austria

**Keywords:** activities of daily living, physiotherapy, quality of life, robot-assisted gait training, stroke, walking

## Abstract

**Introduction:**

The effectiveness of robot-assisted gait training (RAGT) after stroke remains uncertain, particularly regarding its role across different recovery stages. This single-blind, randomized, parallel-group trial examined whether a 4-week inpatient program of end-effector-based RAGT plus conventional physiotherapy (RAGT+CPT) improved walking ability and basic activities of daily living (ADL) more than conventional physiotherapy alone (CPT), and whether effects differed by stroke phase.

**Methods:**

One hundred people with subacute (>7 days to ≤6 months) or chronic (>6 months) stroke were stratified by stroke phase and randomly allocated (1:1) to RAGT+CPT or CPT. Interventions were delivered four times weekly for 4 weeks (45–50 min RAGT/CPT + 25 min CPT). Primary outcomes were walking ability (Functional Ambulation Categories) and basic ADL (Barthel Index). Secondary outcomes included walking speed and distance, functional mobility, concerns about falling, fatigue, health-related quality of life (HRQoL), intervention acceptability, and RAGT gait parameters. Outcomes were assessed at baseline, post-intervention, and follow-up (Week 12). Analyses followed the intention-to-treat principle.

**Results:**

Median (range) baseline FAC was 4 (RAGT+CPT: 0–4; CPT: 1–4) in the subacute groups and 3 (RAGT+CPT: 0–4; CPT: 1–4) in the chronic groups. Cumulative link mixed models demonstrated significant improvements over time in walking ability and basic ADL, with no evidence of differential treatment effects between RAGT+CPT and CPT. Improvements in walking endurance were greater in subacute- than chronic-phase participants. Additional analyses adjusting for baseline ambulatory function did not materially alter the intervention-related conclusions. Self-perceived HRQoL assessed with the EQ-5D-5L Visual Analogue Scale improved significantly more following RAGT+CPT than CPT, whereas most other secondary outcomes were comparable between interventions. No study-related serious adverse events occurred.

**Discussion:**

This trial found no clear superiority of RAGT+CPT over CPT for walking ability or basic ADL. Both interventions produced meaningful improvements over time across both stroke phases, supporting intensive inpatient rehabilitation beyond the early recovery phase. Although objective functional outcomes were comparable between interventions, RAGT+CPT was associated with greater improvement in self-perceived HRQoL. Overall, RAGT may be considered a complementary rehabilitation approach rather than a universally superior alternative to conventional physiotherapy.

**Clinical trial registration:**

https://drks.de/search/en/trial/DRKS00029910/details, identifier DRKS00029910.

## 1 Introduction

After a stroke, walking impairment is a frequent and disabling symptom (1). Approximately 75% people with a stroke experience a walking disorder (2, 3) affecting mobility, balance, and coordination and limiting activities of daily living (ADL) and social participation (4, 5). Consequently, such people are often unable to work (6). Conventional physiotherapy (CPT), which includes overground gait training, stair climbing, and treadmill training, is widely used in post-stroke rehabilitation, underpinning significant improvements in walking speed, endurance, ADL, participation, and quality of life (7, 8). In recent years, robot-assisted gait training (RAGT) has been introduced to complement CPT. It aims to reduce dependence on physiotherapists and lessen the physical effort required to position paretic limbs and manage weight shifts, which can limit therapy intensity, particularly in people with severe impairments (9). RAGT is based on the principle that task-specific, repetitive training supports motor learning and facilitates functional recovery (10, 11). It includes both end-effector and exoskeleton systems (12). During end-effector-based RAGT, feet are positioned on two footplates, simulating the stance and swing phases of gait. Recent research suggests that end-effector systems offer several advantages over exoskeletons, including quicker setup (13), customisation for specific protocols, diseases, and ages (13, 14), greater physical activity (15), and improved outcomes in activity and participation (16).

Evidence from studies investigating RAGT after stroke is mixed, with some trials reporting improvements in walking-related outcomes compared with conventional therapy (17–20) while others have found little or no additional benefit (21–24). Recent meta-analyses suggest that RAGT may support improvements in walking after stroke; however, clear superiority over conventional rehabilitation has not been consistently demonstrated (25). Importantly, this evidence base is characterized by substantial clinical and methodological heterogeneity, including wide variation in stroke chronicity, intervention dose, and comparator treatments. A Cochrane review similarly highlighted the need for well-designed, adequately powered trials to clarify the benefits and potential harms of electromechanical-assisted gait training for walking after stroke (9). In particular, uncertainty remains regarding whether responsiveness to RAGT differs according to the time elapsed since the stroke. Although limited evidence suggests that people in the early post-stroke phases may benefit more than those in the chronic phase, these finding remain inconclusive and require further investigation (26). Moreover, many previous trials have focused primarily on walking outcomes, with comparatively limited attention to ADL, falls, functional mobility, concerns about falling, health-related quality of life (HRQoL), fatigue, and the sustainability of effects beyond the immediate intervention period (4, 9, 27–29). Outcomes related to perceived exertion, acceptability, and training performance—factors that are critical for clinical implementation—are also infrequently reported.

This randomized controlled trial aimed to compare the effects of a time-matched 4-week combined RAGT+CPT program with those of a 4-week CPT program within an inpatient multidisciplinary rehabilitation setting, and to explore whether responses differed according to stroke phase (subacute versus chronic). Outcomes included walking ability, basic ADL, walking speed and distance, falls, functional mobility, concerns about falling, HRQoL, and fatigue. In addition, perceived exertion, intervention acceptability, and gait parameters during RAGT were examined. We hypothesized that both interventions would be associated with improvements over time across functional and patient-reported outcomes in participants with subacute and chronic stroke. We further hypothesized that treatment responses may differ according to intervention type and stroke phase, with the possibility of greater responsiveness to RAGT during the subacute phase.

## 2 Materials and methods

### 2.1 Design and ethical approval

This study was an assessor-blinded single center randomized controlled trial and is reported in accordance with the Consolidated Standards of Reporting Trials (CONSORT) statement (30) (Supplementary Checklist S1). This clinical investigation further followed the World Medical Association Declaration of Helsinki - Ethical Principles for Medical Research involving human subjects (2013) (31), Guidelines of the International Organization for Standardization (ISO), ICH E6 Guideline for Good Clinical Practice (2016) and Guideline for Clinical investigation of medical devices for human subjects - Good clinical practice (ISO/ DIS 14155:2018). The study protocol was approved by the research ethics committee of the Medical University of Innsbruck, Austria (ref. 1245/2022; 07/10/2022) and prospectively registered with the German Clinical Trials Register on 17/10/2022 (ID: DRKS00029910)^1^; subsequent amendments (25/05/2023) were approved by the ethics committee and updated in the trial register. Participants provided informed written consent prior to participation.

### 2.2 Participants

People with subacute or chronic stroke were recruited between 18/10/2022 and 17/03/2025 from the Clinic for Rehabilitation Muenster, Austria and randomly allocated using stratified randomization according to stroke phase. Sample size estimation was performed in G*Power 3.1.9.4 (32) using a repeated-measures ANOVA (within–between interaction) with four between-subject groups (2 intervention groups × 2 strata) and two repeated measurements. Assuming *α* = 0.025, power = 80%, and a conservative small-to-medium effect size (Cohen’s *f* = 0.20), a total sample of 88 participants was required. The selected effect size was informed by the available evidence from the Cochrane review of electromechanical-assisted gait training after stroke (9) and was chosen conservatively to avoid overestimating treatment effects in the anticipated heterogeneous study population with varying baseline walking abilities. To account for an anticipated dropout rate of 12%, the target sample size was increased to 100 participants (50 per group; 25 per stratum).

The inclusion criteria were: adult people (≥18 years of age) with a first-ever ischaemic or haemorrhagic stroke (33, 34) in either the subacute (between 7 days and 6 months post-stroke) or chronic phase (>6 months post-stroke; no upper limit) (35) of any gender identity (female, male, or diverse); from any ethnic background; fluent in German (both spoken and written); with gait impairment, confirmed by a neurologist and a Functional Ambulation Categories (FAC) score of 0–4/5 (36). People with severe subacute or chronic stroke who were unable to walk were eligible if they could sit unsupported (without holding onto a stabilizing object), with feet supported, and maintain an upright sitting position for at least 1 hour, twice daily. They also had to understand the patient information, study procedures, and follow instructions, demonstrated by a Mini Mental Status Test (MMST) score of ≥21/30 (37). Additionally, the principal investigator had to deem them capable of completing the full trial duration.

Exclusion criteria comprised medical, neurological, orthopedic, cardiovascular, or psychiatric conditions that could limit safe participation in active rehabilitation or interfere with the intervention. These included conditions affecting musculoskeletal integrity, vascular status, skin integrity, bone health, spasticity, or sensorimotor function of the lower extremities; contraindications related to cardiovascular or seizure risk; pregnancy; recent medication changes affecting gait or functional performance; and failure to meet device-specific height or weight limits (for details, see Supplementary File S1).

### 2.3 Randomization and blinding

**Figure 1:**
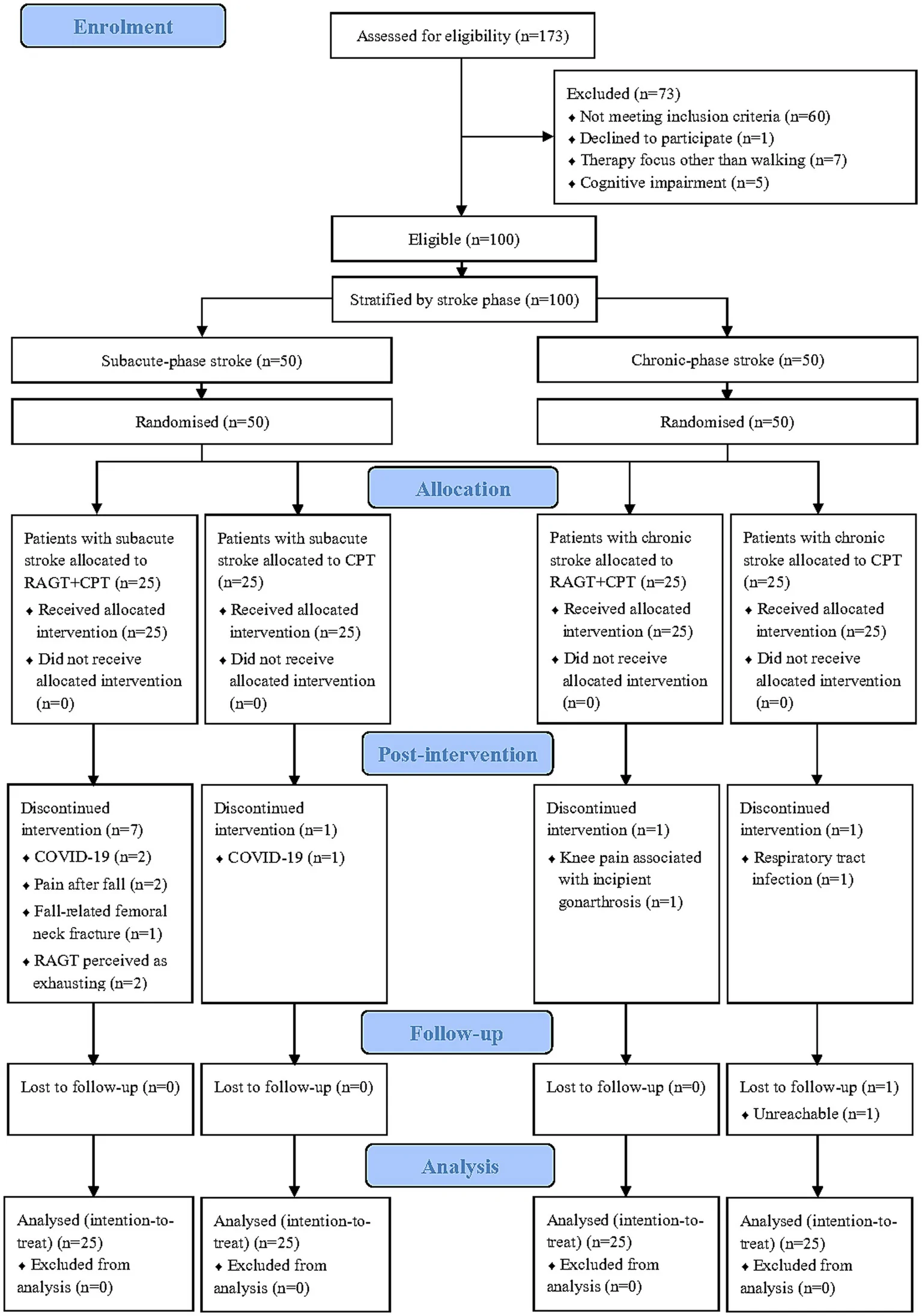
CONSORT flow diagram of this study. Participants were stratified by stroke phase (subacute vs. chronic) prior to randomization to robot-assisted gait training plus conventional physiotherapy (RAGT+CPT) or conventional physiotherapy alone (CPT).

Stroke phase (subacute or chronic) was determined at screening. Participants were then randomly allocated in a 1:1 ratio to RAGT+CPT or CPT using stratified block randomization according to stroke phase, with an online-generated allocation sequence and permuted blocks of concealed size (38). This resulted in four analytic groups defined by stroke phase and intervention (Figure 1). Allocation concealment was ensured by an independent researcher who was not involved in recruitment, intervention delivery, or outcome assessment, and who prepared opaque, sequentially numbered, sealed envelopes. Following eligibility screening and obtaining written informed consent, participants were asked to open the envelope to determine intervention allocation. Participants were not informed of the study hypotheses and outcome assessors remained blinded to group allocation throughout the study.

### 2.4 Intervention

Participation lasted approximately 3 months (4-week intervention, 8-week follow-up). Participants with subacute stroke (Groups 1–2) or chronic stroke (Groups 3–4) were allocated to combined RAGT plus CPT or to time-matched CPT alone. The combined intervention consisted of RAGT (45–50 min, including setup and rest breaks, 4×/week) plus CPT (25 min, 2×/week) for 4 weeks and was delivered by physiotherapists certified in the use of the robotic device (Groups 1 and 3). The CPT-only intervention (70–75 min per session, same weekly schedule) was provided to participants with subacute stroke (Group 2) or chronic stroke (Group 4) and was supervised by experienced physiotherapists. Recorded session time included set-up, safety checks, transfers, rest breaks and active practice (see Supplementary Checklist S2, Template for Intervention Description and Replication [TIDieR]). Movement repetitions (e.g., step counts) were not matched between arms. Perceived exertion was monitored every 7–10 min using the Borg Category Ratio (CR10) scale (39) to guide progression of training intensity and ensure a safe, tolerable workload; it was not used to standardise dose. Rest breaks were available ad libitum.

In addition to the study interventions, all participants received standard inpatient multidisciplinary rehabilitation care as part of routine clinical practice. This included medical management and, where clinically indicated, adjunct therapies such as electrotherapy, respiratory therapy, speech and language therapy, psychological support, dietetic counseling, and structured group activities (e.g., singing or inhalation therapy). These services were delivered according to individual rehabilitation plans and were comparable across intervention groups.

#### 2.4.1 Conventional physiotherapy

CPT followed a standard protocol of individualized, therapist-supervised gait and balance training delivered face-to-face. Content included overground gait training, stair negotiation, transfer practice, and balance tasks in seated and standing, with or without parallel bars and/or a walking aid, as indicated. Physiotherapists initially assisted paretic knee control and transfers and gradually tapered assistance as performance improved. Training was progressed by reducing manual assistance and reliance on parallel bars or walking aids, increasing task complexity (e.g., uneven surfaces and dual-task activities), and advancing step length, walking speed, and walking distance. Progression followed predefined difficulty bands when participants achieved the target perceived exertion (Borg CR10: 3–5) (40)) while maintaining adequate knee control and foot clearance. Therapists provided explicit feedback on performance; implicit feedback was provided by task demands and distance walked.

#### 2.4.2 Robot-assisted gait training

RAGT was delivered using an end-effector system with a dynamic body-weight support harness. Fidelity procedures comprised therapist training on the written standard operating procedure (SOP) and per-session documentation of parameters, mode proportions, Borg ratings and rationale for any deviations. Mode selection and progression were prespecified and applied consistently. Therapists followed an SOP: passive mode was used when participants could not initiate or sustain stepping/weight shift or had inadequate knee control, or when spasticity, pain or fatigue limited active contribution. Transition to assistive mode required consistent active participation and adequate knee control. Within and between sessions, body-weight support, mode proportion, speed/cadence, step length, and pelvic displacement were progressed according to predefined difficulty bands when participants achieved the target perceived exertion [Borg CR10: 3–5 (40)], while maintaining adequate knee control and foot clearance.

Participants began at approximately 1.5 km/h, with gradual increases toward 2.2–2.5 km/h as tolerated. Explicit feedback was provided by the therapist, and implicit augmented feedback (e.g., gait cycle targets, weight bearing, step length, distance, session duration) was displayed on the device interface in real time. Device logs captured gait parameters automatically. The first session was treated as an introductory familiarization and excluded from analyses. For descriptive/exploratory summaries of training progression, data from sessions 2–5, 6–8, 9–12 and 13–16 were aggregated into four consecutive time windows.

#### 2.4.3 Instrument

**Figure 2:**
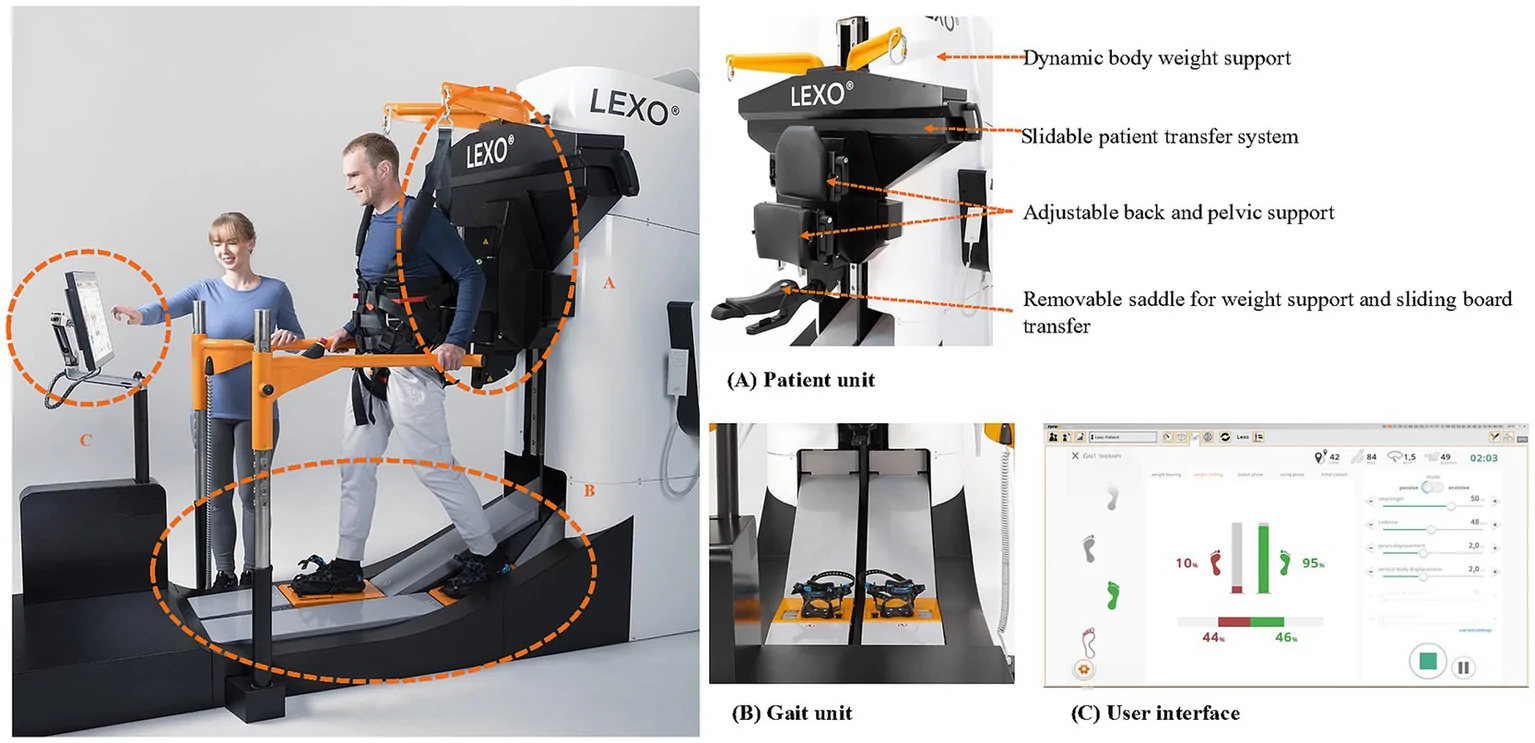
Lexo robotic gait trainer (Tyromotion) used for this study. The images show a patient using a harness suspension system for weight support, with connections at the back and pelvis providing trunk support and pelvic guidance. The highly adaptable hardware and software facilitate demanding self-activity during specific gait training. These highlighted areas: **(A)** Patient unit; **(B)** Gait unit: sensor-equipped adjustable footplates and foot fixation; **(C)** User interface with augmented performance feedback. © Tyromotion GmbH.

Lexo (Tyromotion GmbH, Graz, Austria) is a stationary, end-effector robotic gait trainer commercially available for the rehabilitation of people with impaired walking and mobility. The system comprises three main components: the patient unit, the gait unit, and the user interface (Figure 2). The feet are secured to motorized footplates that guide the gait cycle, while the legs remain unrestrained. The patient unit includes a dynamic, electrically driven body weight support system and a pelvic guidance mechanism. The user interface is a computer-based control system that allows adjustment of therapy parameters during walking. The TyroS software provides a virtual training environment with modular, task-specific exercises targeting gait components such as weight shifting, leg loading, initial contact, and stance and swing phases. Real-time sensor feedback is integrated to monitor performance.

Lexo hardware and software settings can be scaled to different levels of impairment, ranging from patients with FAC 0 to those with FAC 4. Optional attachments at the trunk and pelvis provide additional support and enable guided pelvic movement. The system is designed to promote active participation and allow variability in movement, consistent with principles of motor learning (41, 42). Several features address practical aspects of clinical use, including adaptive assistance, adjustable constraints, real-time feedback, performance monitoring, modular task training, and a simplified interface (43, 44). The end-effector design avoids the need for joint alignment required in exoskeletons, reducing setup times (45). Patient transfer can be facilitated by either a motorized lifter or a sliding board.

### 2.5 Outcomes and safety assessment

Walking and mobility outcomes were assessed face-to-face by the same trained, blinded assessors at baseline (Week 1) and post-intervention (Week 4). During the intervention period, perceived exertion was monitored, and overall acceptability was evaluated post intervention. Questionnaire-based outcomes assessing concerns about falling, HRQoL, and fatigue were completed face-to-face at Weeks 1 and 4, and at Week 12 follow-up via telephone. Intervention adherence was documented throughout the intervention period.

Falls were defined according to the Prevention of Falls Network Europe (ProFaNE) consensus (46). At baseline, participants reported falls occurring during the 4 weeks prior to enrolment by recall. Thereafter, falls were recorded prospectively by participants using a falls diary during the 4-week intervention period and the subsequent 8-week follow-up. Outcomes were assessed as (i) the total number of falls and (ii) the proportion of participants classified as fallers (≥1 fall) versus non-fallers across these three time periods. Adverse events (AE) and side effects were recorded in a formal log, noting type, dates, severity, causality (serious/non-serious), actions taken, and outcomes. Study-related events, such as training injuries, were treated at no cost. Severe AEs would have resulted in early termination of the investigation. Monitoring and auditing procedures were performed throughout the study independently from the device manufacturer.

#### 2.5.1 Primary outcomes

Primary outcomes were walking ability, assessed using the FAC (36, 47) and basic ADL, measured with the Barthel Index (BI) (48). The validated German versions of both scales were used (49, 50). Walking ability was screened and evaluated post-intervention using the German FAC. An assessor posed brief questions and observed walking to assign a score from 0 (non-functional ambulator) to 5 (independent on all surfaces). Scores of 1–3 denote dependence on another person: continuous manual contact (1), intermittent/continuous contact (2), or verbal supervision (3). A score of 4 indicates independent walking on level surfaces only (51). A 1-point change was considered clinically significant (52).

Basic ADL were assessed with the German BI, which also indicates care needs. The 10-item scale includes feeding, bathing, grooming, dressing, toileting, bladder/bowel control, transfers, walking, and stair use. Items are weighted by required support and scored as 0 (dependent), 5 (some help), or 10 (independent), with a maximum score of 100 (48). As no validated MCID for the 100-point Barthel Index was identified, no MCID threshold was applied.

#### 2.5.2 Secondary outcomes

Secondary outcomes included walking speed, assessed by the 10-Meter Walk Test (10MWT) (53), walking distance, measured by the 6-Minute Walk Test (6MWT) (54), and functional mobility, evaluated using the Timed Up and Go (TUG) test (55) and the Rivermead Mobility Index (RMI) (56). Concerns about falling were measured using the Falls Efficacy Scale-International (FES-I) (57), HRQoL was evaluated using the Short Form Stroke Impact Scale (SF-SIS) (58) and the 5-level EQ-5D (EQ-5D-5L) (59), and fatigue was assessed with the Fatigue Severity Scale (FSS) (60). A Smiley Likert Scale assessed intervention acceptability (61). Detailed description of the assessments is provided in the Supplementary File S2.

### 2.6 Data analyses

Data analyses were conducted using IBM SPSS Software, Release 30.0 (IBM, Armonk, New York, USA) and GraphPad Prism 10 (Boston, Massachusetts USA). No interim analyses were planned or conducted for this trial. Statistical significance was set at a two-sided *p*-value of less than 0.05. Missing data were examined before analysis. For isolated missing observations assumed to be missing at random (e.g., missed assessments or unavailable measurements), multiple imputation was performed in SPSS using the fully conditional specification (chained equations) method. The imputation model included relevant baseline characteristics and outcome measures. Participants who were unable to perform the walking-based assessments because of their functional limitations were not considered to have missing outcome data. For the 10MWT and 6MWT, values of 0 m/s and 0 m, respectively, were assigned to indicate inability to perform the assessment. For the TUG, a coding value of 0 was assigned to indicate inability to perform the assessment, as a completion time could not be obtained. If participants subsequently became able to perform a walking assessment, the observed test value was recorded. These predefined values represented observed outcomes and were therefore not included in the multiple imputation procedure. All analyses were conducted according to the intention-to-treat principle, with participants analyzed in the groups to which they were originally randomized, irrespective of adherence to the allocated intervention. For clinical outcomes, following normality tests on continuous data, descriptive statistics (mean and standard deviation or median and range) were calculated per assessment. Baseline group comparisons were conducted using One-Way ANOVA, Kruskal-Wallis, and Chi-squared tests.

To examine whether the imbalance in baseline ambulatory function influenced the findings, *post hoc* sensitivity analyses were conducted for the continuous walking outcomes using linear mixed-effects models. Separate models were fitted for the 6MWT, 10MWT, and TUG. Intervention group, stroke phase, time, all corresponding two-way interactions, and the intervention group × stroke phase × time interaction were included as fixed effects. Baseline FAC was entered as a numeric covariate, reflecting its ordered 0–5 scale; this specification assumes an approximately linear association between FAC and the continuous outcome. Participant ID was specified as the subject variable, and the correlation between repeated observations was modeled using a compound-symmetry covariance structure. Models were estimated using restricted maximum likelihood, and Type III tests of fixed effects were reported. Estimated marginal means and 95% confidence intervals were calculated at the mean baseline FAC value. These sensitivity analyses used all available repeated observations; genuinely missing outcome measurements were not imputed for the mixed-model analyses. The analyses were conducted *post hoc* to assess the robustness of the prespecified primary findings and did not replace the primary mixed-ANOVA analyses. The *post hoc* linear mixed-model sensitivity analyses used all available repeated observations under the missing-at-random assumption. In contrast to the primary analyses, no additional multiple imputation was performed for genuinely missing repeated outcome measurements in these sensitivity analyses.

Ordinal outcomes were analyzed using cumulative link mixed models (CLMMs) after collapsing scale scores into clinically interpretable ordered categories. This approach was chosen because several outcomes were derived from ordinal clinical scales with numerous sparse score levels, making models based on the original score distributions numerically unstable. Categories were defined *a priori* using established or clinically meaningful cut-points where available. The FAC was categorized as 0–3 (dependent/supervised ambulation), 4 (limited community ambulation), and 5 (independent community ambulation) (51, 62). Because no participants were classified as FAC 5 at baseline (an exclusion criterion), an initial dichotomous model (FAC 0–3 vs. 4–5) (51, 62) resulted in quasi-complete separation and unstable parameter estimates. A three-category model was therefore adopted, as it retained clinically meaningful distinctions while providing stable model estimation; the BI as 0–60 (severely dependent), 61–90 (moderately dependent), and 91–100 (slightly dependent to independent) (63); the FES-I as 16–19 (low concern), 20–27 (moderate concern), and 28–64 (high concern about falling) (64, 65); the FSS as 9–35 (low/no fatigue), 36–44 (moderate fatigue), and 45–63 (severe fatigue) (66, 67); RMI, SF-SIS and EQ-5D-5L VAS scores were categorized into tertiles based on the distribution of scores in the study population (68).

For each ordinal outcome, a cumulative logit mixed model with a participant-specific random intercept was fitted to account for repeated measurements. Fixed effects included time, treatment group, their interaction, and stroke stage as an adjustment covariate. The primary treatment-effect test was the Time × Treatment Group interaction, which assessed whether change over time differed between treatment groups. Stroke stage was included as an adjustment covariate but was not interacted with time or treatment group because preliminary models including the three-way interaction (Time × Treatment Group × Stroke Stage) produced unstable parameter estimates owing to sparse outcome categories and the available sample size. Outcomes available at three time points were analyzed across baseline, post-intervention, and follow-up, whereas FAC and BI were analyzed across baseline and post-intervention only. Unused time levels were removed separately for each outcome before model fitting. Regression coefficients were exponentiated and reported as odds ratios (ORs) with 95% confidence intervals (CIs). Overall effects of the fixed terms were assessed using Wald χ^2^ tests. Where significant interactions were identified, Holm-adjusted *post hoc* comparisons based on estimated marginal means were performed.

Models were fitted in R (R Foundation for Statistical Computing, Vienna, Austria) (69) using the clmm() function from the ordinal package. Estimated marginal means and *post hoc* contrasts were obtained using the emmeans package. Data handling and tabulation were performed using the dplyr, tibble, and rlang packages, and results were exported using writexl. Model adequacy was assessed using convergence diagnostics, including the maximum absolute gradient and positive-definiteness of the Hessian. Models showing inadequate convergence, non-positive-definite Hessians, or implausibly large standard errors were not interpreted.

For continuous outcomes, data were analyzed using a three-way mixed-design analysis of variance (ANOVA). Time (baseline, post-intervention; and where applicable follow-up) was modeled as the within-subject factor, while intervention group (RAGT+CPT vs. CPT) and stroke phase (subacute vs. chronic) were included as between-subject factors. This model allowed the examination of main effects of time, intervention, and stroke phase, as well as two-way (time × intervention; time × stroke phase) and three-way (time × intervention × stroke phase) interaction effects. The assumption of sphericity for within-subject effects was assessed using Mauchly’s test. Where sphericity was violated (*p* < 0.05), Greenhouse–Geisser corrections were applied to adjust the degrees of freedom. *Post hoc* analyses with Bonferroni adjustment were conducted where appropriate to support interpretation of significant interaction effects.

## 3 Results

As detailed in the flow diagram (Figure 1), 173 people after stroke were screened for eligibility; 73 did not meet the inclusion criteria and were excluded. The study enrolled 100 participants, with 50 in the subacute and 50 in the chronic stroke categories. Participants were randomized to receive RAGT+CPT (Groups 1 and 3, *n* = 25 each) or CPT alone (Groups 2 and 4, *n* = 25 each). In Group 1 (subacute RAGT+CPT), seven participants withdrew owing to health issues (five unrelated and two possibly related to RAGT-related fatigue). Groups 2–4 each had one withdrawal (two unrelated and one possibly related to knee pain in Group 3). All randomized participants were included in the intention-to-treat analysis in their originally allocated groups. RAGT+CPT groups received a mean of 15 (median 16, range 12–16) RAGT sessions plus 8 CPT sessions focused on gait training. CPT-only participants had sixteen 45–50-min and eight 25-min CPT sessions, all targeting gait training.

### 3.1 Baseline characteristics

The intention-to-treat sample included 62 men and 38 women, with a mean (± standard deviation) age of 67.90 ± 11.72 years. Eighty-three had experienced an ischaemic stroke and 17 a haemorrhagic stroke. Among those with subacute stroke, the mean time since onset was 0.27 ± 0.17 years, compared with 8.07 ± 13.27 years in those with chronic stroke. At baseline, demographic and disease-related characteristics were generally comparable between groups (Table 1). Despite randomization, some imbalance in baseline ambulatory function was observed, with a greater proportion of participants with FAC scores of 0–1 and unable to perform the walking assessments in the RAGT+CPT group.

**Table 1:** Baseline characteristics of the intention-to-treat sample.

Parameter	RAGT+CPT subacute stroke (*n* = 25)	CPT subacute stroke (*n* = 25)	RAGT+CPT chronic stroke (*n* = 25)	CPT chronic stroke (*n* = 25)
Sex (n, %)				
Male	12 (12.0)	17 (17.0)	17 (17.0)	16 (16.0)
Female	13 (13.0)	8 (8.0)	8 (8.0)	9 (9.0)
Age (years)^a^	70.2 ± 10.9	71.4 ± 10.2	64.4 ± 11.4	65.6 ± 13.3
Etiology (*n*, %)				
Ischaemic stroke	21 (21.0)	21 (21.0)	21 (21.0)	20 (20.0)
Haemorrhagic stroke	4 (4.0)	4 (4.0)	4 (4.0)	5 (5.0)
Time since stroke (years)^a^	0.2 ± 0.2	0.3 ± 0.2	9.6 ± 16.0	6.5 ± 9.9
NIHSS^b^	7 (4–22)	6 (3–15)	8 (3–15)	6 (3–12)
MMST^b^	29 (24–30)	28 (23–30)	29 (21–30)	30 (21–30)
BI^b^	90 (15–100)	85 (35–100)	80 (25–100)	80 (40–100)
FAC 0 (*n*, %)	6 (6.0)	0 (0)	1 (1.0)	0 (0)
FAC 1 (*n*, %)	1 (1.0)	1 (1.0)	5 (5.0)	3 (3.0)
FAC 2 (*n*, %)	0 (0)	2 (2.0)	2 (2.0)	4 (4.0)
FAC 3 (*n*, %)	3 (3.0)	6 (6.0)	6 (6.0)	8 (8.0)
FAC 4 (*n*, %)	15 (15.0)	16 (16.0)	11 (11.0)	10 (10.0)

a Mean ± standard deviation (SD).

b Median (range).

BI, Barthel Index, CPT, conventional physiotherapy; FAC, Functional Ambulation Categories; MMST, Mini Mental Status Test; NIHSS, National Institute of Health Stroke Scale; RAGT, robot-assisted gait training.

### 3.2 Adverse events

Adverse events were generally mild and comparable across groups. Thirty-four adverse events were reported in 31 participants, including one serious adverse event (fall-related femoral neck fracture), which was judged unrelated to the intervention. Three mild adverse events were considered possibly related to RAGT. No severe intervention-related adverse events occurred. Detailed adverse event data are provided in the Supplementary Table S1 (89).

### 3.3 Primary outcomes

**Figure 3:**
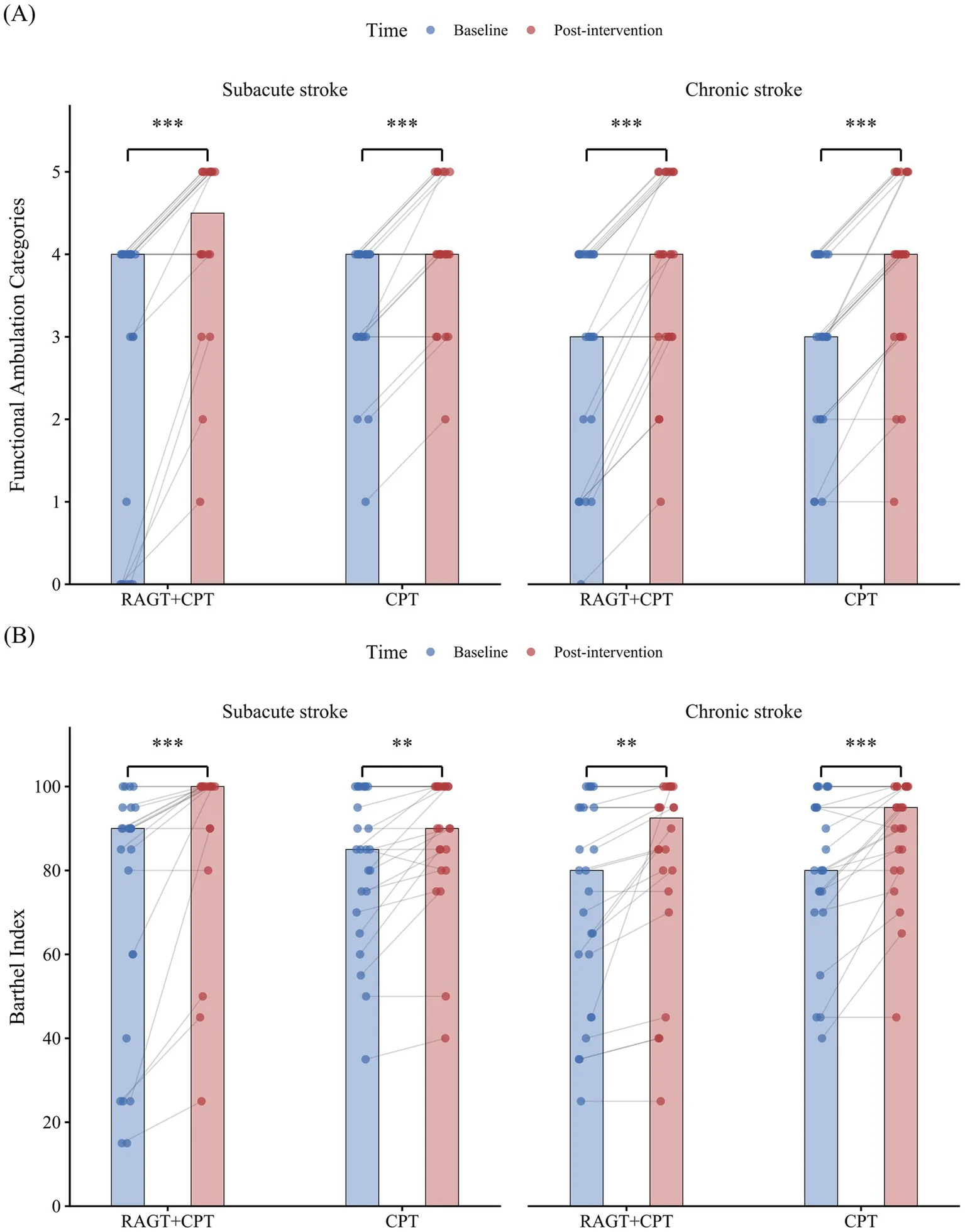
Changes in walking ability and basic activities of daily living from baseline to post-intervention by treatment group and stroke stage. **(A)** Functional Ambulation Categories (FAC) and **(B)** Barthel Index (BI) scores are shown for participants with subacute and chronic stroke, separately for RAGT+CPT and CPT. In the paired plots, blue points and bars represent baseline, and red points and bars represent post-intervention. Individual points represent participant-level observations, bars represent group summary values, and gray lines connect repeated measurements within participants. Statistical analyses were performed using cumulative link mixed models (CLMMs) based on collapsed FAC and BI categories, with fixed effects for time, treatment group, the Time × Treatment interaction, and stroke stage, and a participant-specific random intercept. Brackets indicate model-based within-group comparisons between baseline and post-intervention within each treatment group and stroke-stage stratum. Higher FAC scores indicate greater walking independence, and higher BI scores indicate greater independence in activities of daily living. Significance levels are indicated as *p < 0.05; **p < 0.01; ***p < 0.001. FAC, Functional Ambulation Categories; BI, Barthel Index; RAGT+CPT, robot-assisted gait training plus conventional physiotherapy; CPT, conventional physiotherapy.

The FAC model, categorized as FAC 0–3, FAC 4, and FAC 5, included 190 observations from 100 participants across baseline and post-intervention and converged satisfactorily. In the RAGT+CPT reference group, the odds of being in a higher FAC category were significantly greater post-intervention than at baseline (OR = 42.40, 95% CI 6.57–273.57; *p* < 0.001), indicating greater odds of achieving a higher level of walking independence over time. At baseline, FAC category did not differ significantly between the CPT and RAGT+CPT groups (OR = 1.23, 95% CI 0.21–7.08; *p* = 0.816). Stroke stage was not significantly associated with FAC category (OR = 0.28, 95% CI 0.05–1.43; *p* = 0.126), and the Time × Treatment interaction was not significant (OR = 0.60, 95% CI 0.12–2.90; *p* = 0.524), indicating no evidence that changes in FAC category differed between treatment groups. Omnibus Wald tests confirmed a significant main effect of time (χ^2^(1) = 18.73, *p* < 0.001), whereas treatment group, stroke stage, and the Time × Treatment interaction were not significant (all *p* > 0.05). Descriptively, the number of participants in FAC 0–3 decreased from 48 at baseline to 25 post-intervention, whereas the number classified as FAC 5 increased from 0 to 30 (Figure 3). Descriptive statistics for the primary outcomes at baseline and post-intervention, and within-group changes over time are presented in the Supplementary Table S2.

The collapsed BI model included 190 observations from 100 participants across baseline and post-intervention assessments and converged satisfactorily. In the RAGT+CPT reference group, the odds of being in a better Barthel Index dependency category were significantly higher post-intervention than at baseline (OR = 28.54, 95% CI 2.90–281.08; *p* = 0.004), indicating improved functional independence over time. At baseline, the CPT group did not differ significantly from the RAGT+CPT group (OR = 7.28, 95% CI 0.52–101.20; *p* = 0.139), and stroke stage was not significantly associated with BI category (OR = 1.34, 95% CI 0.14–13.28; *p* = 0.802). The Time × Treatment interaction was not statistically significant (OR = 0.22, 95% CI 0.03–1.82; *p* = 0.160), indicating no evidence that changes in BI category differed between treatment groups. Omnibus Wald tests confirmed a significant main effect of time on BI category (χ^2^(1) = 10.55, *p* = 0.001), whereas treatment group, stroke stage, and the Time × Treatment interaction were not statistically significant (all *p* > 0.05). Descriptively, the proportion of participants classified as having mild or no dependency increased from baseline to post-intervention in both treatment groups (Supplementary Table S2).

**Figure 4:**
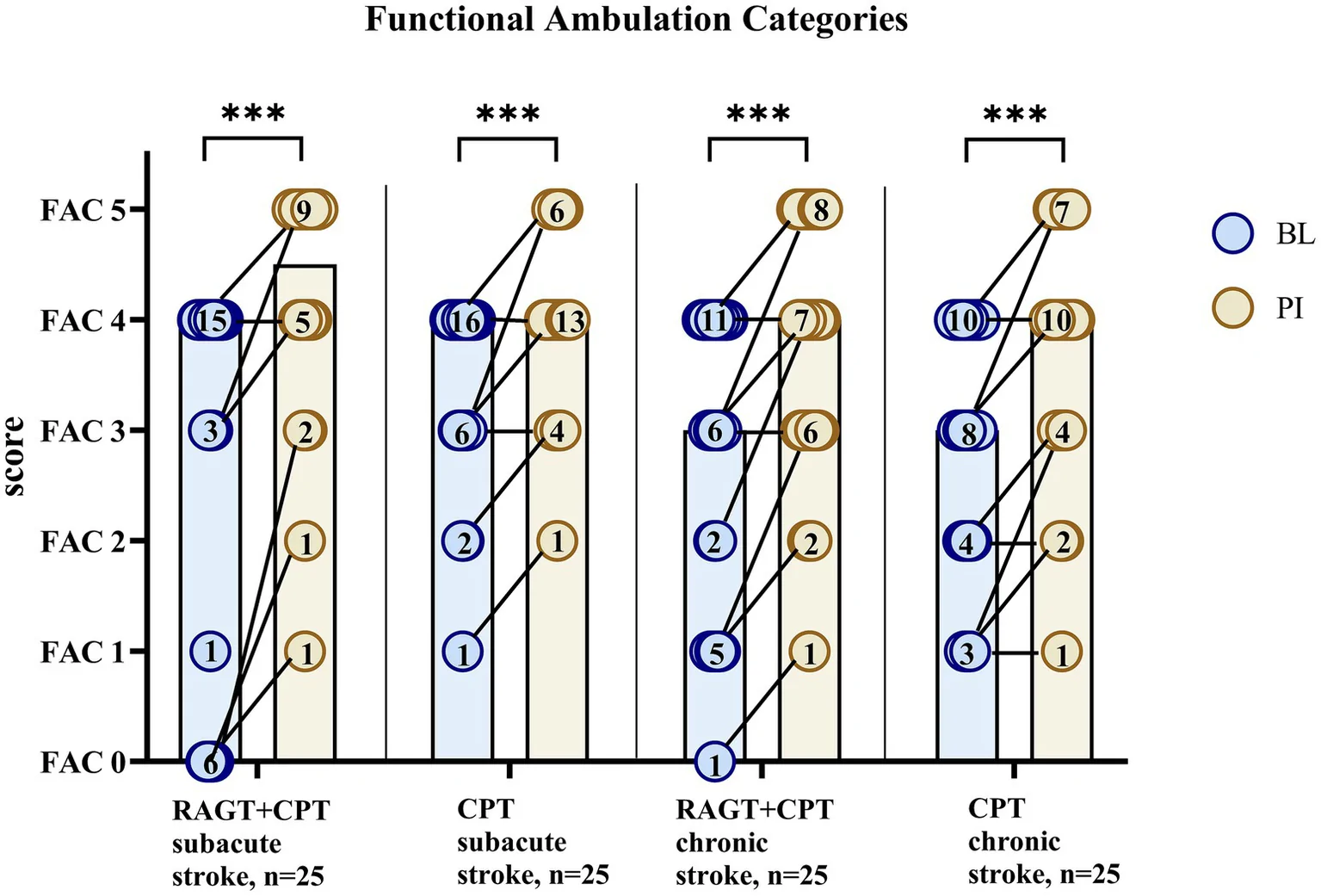
Individual changes in walking ability across groups. The scatter dot plot displays individual participants’ walking ability at baseline (Week 0, blue dots) and post-intervention (Week 4, ochre dots), assessed using the Functional Ambulation Categories (FAC), where higher scores indicate better walking function. Numbers within the dots indicate the number of participants with each FAC score. Vertical bars represent the median; ns, not significant; *p < 0.05; **p < 0.01; ***p < 0.001. RAGT, robot-assisted gait training; BI, baseline; CPT, conventional physiotherapy; PI, post-intervention.

Among participants who were dependent walkers at baseline (FAC < 4), 2/25 (8.0%) in the subacute RAGT+CPT group and 4/25 (16.0%) in the chronic RAGT+CPT group achieved independent walking (FAC ≥ 4) after the intervention, compared with 4/25 (16.0%) and 8/25 (32.0%) participants, respectively, in the corresponding CPT groups. Following RAGT+CPT, 12/25 (48%) subacute-phase and 8/25 (32%) chronic-phase participants demonstrated an improvement of at least one FAC level, whereas after CPT alone, 8/25 (32%) subacute-phase and 11/25 (44%) chronic-phase participants showed a comparable improvement (Figure 4).

### 3.4 Secondary outcomes

#### 3.4.1 Functional assessments - changes from week 0 to week 4

At baseline, 25 participants (25.0%) were unable to perform the 10MWT, 6MWT, and TUG (subacute RAGT+CPT, 7/25 [28%]; subacute CPT, 3/25 [12%]; chronic RAGT+CPT, 8/25 [32%]; chronic CPT, 7/25 [28%]). This decreased to 10 participants (10.0%) post-intervention (3/25 [12%], 1/25 [4%], 3/25 [12%], and 3/25 [12%], respectively). For walking speed (10MWT), no significant time × intervention interaction was found, *F*(1, 73) = 0.24, *p* = 0.629, *η^2^ₚ* = 0.003. However, a significant time × stroke phase interaction was observed, *F*(1, 73) = 7.63, *p* = 0.007, *η^2^ₚ* = 0.095, indicating greater improvements over time in participants with subacute stroke compared with those in the chronic phase. The three-way interaction (time × intervention × stroke phase) was not statistically significant, *F*(1, 73) = 0.29, *p* = 0.590, *η^2^ₚ* = 0.004. A significant and large main effect of time was observed, *F*(1, 73) = 102.55, *p* < 0.001, *η^2^ₚ* = 0.584, indicating improvements in walking speed from baseline to post-intervention across all participants. Exploratory post-hoc comparisons stratified by stroke phase showed that, among participants with subacute stroke, walking speed improved more following RAGT+CPT than CPT alone (mean difference 0.234 m/s, 95% CI 0.007 to 0.461, *p* = 0.043). In contrast, no significant between-intervention difference was observed in participants with chronic stroke (mean difference −0.096 m/s, 95% CI − 0.352 to 0.133, *p* = 0.405).

For walking distance (6MWT), a significant three-way interaction (time × intervention × stroke phase) phase was observed, *F*(1, 81) = 4.54, *p* = 0.036, *η^2^ₚ* = 0.053, indicating that changes in walking endurance over time differed according to both intervention type and stroke phase. There was no significant time × intervention interaction (*p* = 0.572) or time × stroke phase interaction (*p* = 0.205). Simple-effects analyses showed that within the RAGT+CPT group, participants in the subacute phase achieved significantly greater improvements in walking distance than those in the chronic phase (mean difference 119.6 m, 95% CI 34.9 to 204.2, *p* = 0.006, *η^2^ₚ* = 0.089), whereas no significant subacute–chronic difference was observed within the CPT group (mean difference 52.2 m, 95% CI − 24.4 to 128.9, *p* = 0.179). A significant and large main effect of time was also observed, *F*(1, 81) = 102.87, *p* < 0.001, *η^2^ₚ* = 0.559, reflecting overall improvements in walking distance from baseline to post-intervention.

For functional mobility (TUG), no significant time × intervention interaction was observed, *F*(1, 63) = 0.50, *p* = 0.482, *η^2^ₚ* = 0.008, nor was a significant time × stroke phase interaction detected, *F*(1, 63) = 0.56, *p* = 0.455, *η^2^ₚ* = 0.009. The three-way interaction (time × intervention × stroke phase) was also not statistically significant, *F*(1, 63) = 0.88, *p* = 0.351, *η^2^ₚ* = 0.014. A significant and large main effect of time was observed, *F*(1, 63) = 20.17, *p* < 0.001, η^2^ₚ = 0.242, indicating overall improvements in functional mobility from baseline to post-intervention across participants (Table 2).

**Table 2:** Effect of the intervention on walking speed (10MWT), walking distance (6MWT), and functional mobility (TUG).

Parameter	RAGT+CPT subacute stroke (*n* = 25)	CPT subacute stroke (*n* = 25)	RAGT+CPT chronic stroke (*n* = 25)	CPT chronic stroke (*n* = 25)	Model effects
BL 10MWT (m/s)^a^	0.85 ± 0.65	0.84 ± 0.41	0.52 ± 0.46	0.62 ± 0.56	time × interv., *p* = 0.629, η^2^ₚ = 0.003 time × phase, *p* = 0.455, η^2^ₚ = 0.095 time × interv. × phase, *p* = 0.590, η^2^ₚ = 0.004 main effect of time, *p* < 0.001, η^2^ₚ = 0.584
Δ PI-BL 10MWT (m/s)^a^	0.26 ± 0.17; p_adj_ < 0.001	0.18 ± 0.20; p_adj_ < 0.001	0.09 ± 0.12; p_adj_ = 0.038	0.13 ± 0.15; p_adj_ = 0.001	
Between-group differences					Mean (95% CI)
Δ (Week 4–0): RAGT+CPT subacute vs. CPT subacute stroke					0.23 (0.01 to 0.46)
Δ (Week 4–0): RAGT+CPT subacute vs. RAGT+CPT chronic stroke					0.49 (0.08 to 0.89)
Δ (Week 4–0): RAGT+CPT subacute vs. CPT chronic stroke					0.47 (0.07 to 0.87)
Δ (Week 4–0): CPT subacute vs. RAGT+CPT chronic stroke					0.29 (−0.08 to 0.66)
Δ (Week 4–0): CPT subacute vs. CPT chronic stroke					0.27 (−0.09 to 0.64)
Δ (Week 4–0): RAGT+CPT chronic vs. CPT chronic stroke					−0.10 (−0.35 to 0.13)

Parameter	RAGT+CPT subacute stroke (*n* = 25)	CPT subacute stroke (*n* = 25)	RAGT+CPT chronic stroke (*n* = 25)	CPT chronic stroke (*n* = 25)	Model effects
BL 6MWT (m)^a^	214.12 ± 153.16	229.36 ± 110.66	151.10 ± 133.89	182.02 ± 157.79	time × interv., *p* = 0.527, η^2^ₚ = 0.004 time × phase, *p* = 0.205, η^2^ₚ = 0.020 time × interv. × phase, *p* = 0.036, η^2^ₚ = 0.053 main effect of time, *p* < 0.001, η^2^ₚ = 559
Δ PI-BL 6MWT (m)^a^	69.50 ± 52.24; p_adj_ < 0.001	41.72 ± 52.31; p_adj_ < 0.001	33.93 ± 21.98; p_adj_ < 0.001	52.98 ± 43.63; p_adj_ < 0.001	
Between-group differences					Mean (95% CI)
Δ (Week 4–0): RAGT+CPT subacute vs. CPT subacute stroke					41.03 (−80.44 to 162.51)
Δ (Week 4–0): RAGT+CPT subacute vs. RAGT+CPT chronic stroke					119.57 (−1.90 to 241.05)
Δ (Week 4–0): RAGT+CPT subacute vs. CPT chronic stroke					93.27 (−28.20 to 214.75)
Δ (Week 4–0): CPT subacute vs. RAGT+CPT chronic stroke					78.54 (−31.49 to 188.58)
Δ (Week 4–0): CPT subacute vs. CPT chronic stroke					52.23 (−57.80 to 162.27)
Δ (Week 4–0): RAGT+CPT chronic vs. CPT chronic stroke					−26.30 (−136.34 to 83.73)

Parameter	RAGT+CPT subacute stroke (*n* = 25)	CPT subacute stroke (*n* = 25)	RAGT+CPT chronic stroke (*n* = 25)	CPT chronic stroke (*n* = 25)	Model effects
BL TUG (s)^a^	15.59 ± 17.20	17.58 ± 7.67	24.72 ± 19.39	19.31 ± 12.81	Time × interv., *p* = 0.482, η^2^ₚ = 0.008 Time × phase, *p* = 0.455, η^2^ₚ = 0.009 Time × interv. × phase, *p* = 0.351, η^2^ₚ = 0.014 main effect of time, *p* < 0.001, η^2^ₚ = 242
Δ PI-BL TUG (s)^a^	−10.43 ± 17.78 p_adj_ = 0.0022	−5.95 ± 12.29 p_adj_ = 0.0459	−3.10 ± 3.21 p_adj_ = 0.0980	−3.43 ± 5.58 p_adj_ = 0.0472	
Between-group differences					Mean (95% CI)
Δ (Week 4–0): RAGT+CPT subacute vs. CPT subacute stroke					−2.59 (−14.20 to 9.03)
Δ (Week 4–0): RAGT+CPT subacute vs. RAGT+CPT chronic stroke					−6.19 (−18.70 to 6.31)
Δ (Week 4–0): RAGT+CPT subacute vs. CPT chronic stroke					−4.50 (−16.64 to 7.65)
Δ (Week 4–0): CPT subacute vs. RAGT+CPT chronic stroke					−3.61 (−14.98 to 7.77)
Δ (Week 4–0): CPT subacute vs. CPT chronic stroke					−1.91 (−12.89 to 9.07)
Δ (Week 4–0): RAGT+CPT chronic vs. CPT chronic stroke					1.69 (−10.23 to 13.62)

a Mean ± standard deviation (SD). Higher 10MWT and 6MWT values indicate improvement; lower TUG values indicate improvement. p_adj_ represent adjusted *p*-values (corrected for multiple comparisons). BL, baseline; CI, confidence interval; CPT, conventional physiotherapy; Δ (Delta), difference; interv., intervention; phase, stroke phase; η^2^_p_, partial Eta-squared; PI, post-intervention; RAGT, robot-assisted gait training.

##### 3.4.1.1 Additional analyses adjusting for baseline ambulatory function

To assess whether the baseline imbalance in ambulatory function influenced the primary findings, additional linear mixed-effects analyses were performed for the continuous walking outcomes, adjusting for baseline FAC.

For the 6MWT, adjustment for baseline FAC did not materially alter the findings. The intervention group × stroke phase × time interaction remained statistically significant (*F*(1, 82.52) = 4.09, *p* = 0.046), whereas the time × intervention group and time × stroke phase interactions remained non-significant. The main effect of time also remained significant (*F*(1, 82.50) = 102.84, *p* < 0.001), indicating an overall improvement in walking endurance following the intervention. Baseline FAC was significantly associated with walking distance (*F*(1, 97.25) = 145.60, *p* < 0.001).

For the 10MWT, adjustment for baseline FAC likewise did not alter the intervention-related conclusions. Neither the time × intervention group interaction nor the intervention group × stroke phase × time interaction was statistically significant. However, the previously significant time × stroke phase interaction was no longer statistically significant after adjustment (*F*(1, 85.13) = 0.38, *p* = 0.537). The main effect of time remained significant (*F*(1, 85.14) = 28.93, *p* < 0.001), indicating an overall increase in walking speed from baseline to post-intervention. Baseline FAC was also a significant predictor of walking speed (*F*(1, 93.17) = 136.40, *p* < 0.001), suggesting that differences in baseline ambulatory function contributed to the previously observed difference in improvement between stroke phases.

For the TUG, adjustment for baseline FAC did not materially alter the findings. The time × intervention group, time × stroke phase, and intervention group × stroke phase × time interactions all remained non-significant, whereas the main effect of time remained significant (*F*(1, 60.92) = 19.48, *p* < 0.001), reflecting overall improvement in functional mobility. Baseline FAC was significantly associated with TUG performance (*F*(1, 85.72) = 84.90, *p* < 0.001).

Overall, adjustment for baseline ambulatory function confirmed the robustness of the primary intervention-related findings. Baseline FAC was a significant covariate in all three mixed-effects models, indicating that baseline ambulatory function was an important determinant of performance across all three walking outcomes. The only notable difference was that the previously observed time × stroke phase interaction for walking speed was no longer statistically significant after adjustment for baseline FAC, suggesting that baseline ambulatory function contributed to the apparent difference in recovery between stroke phases. The conclusions regarding the effects of the intervention remained unchanged after adjustment for baseline ambulatory function.

#### 3.4.2 Functional assessments - changes across week 0, week 4, and week 12

At baseline, 35/100 participants (35%) had experienced one or more falls, decreasing to 7/100 (7%) at post-intervention and 7/100 (7%) at follow-up. No significant between-group differences in falls were observed.

For functional mobility assessed with the RMI, the collapsed ordinal model included 278 observations from 100 participants across baseline, post-intervention, and follow-up assessments and converged satisfactorily. In the RAGT+CPT reference group, the odds of being in a higher functional mobility category were significantly higher post-intervention than at baseline (OR = 63.69, 95% CI 4.75–853.83; *p* = 0.002) and at follow-up than at baseline (OR = 39.62, 95% CI 3.09–508.02; *p* = 0.005), indicating improved functional mobility over time. At baseline, the CPT group did not differ significantly from the RAGT+CPT group (OR = 1.59, 95% CI 0.11–22.96; *p* = 0.733), and stroke stage was not significantly associated with RMI category (OR = 1.00, 95% CI 0.07–14.08; *p* = 0.997). The Time × Treatment interactions were not statistically significant at either post-intervention (OR = 0.11, 95% CI 0.006–1.96; *p* = 0.132) or follow-up (OR = 0.31, 95% CI 0.02–5.63; *p* = 0.426), indicating no evidence that changes in RMI category differed between treatment groups. Omnibus Wald tests confirmed a significant main effect of time on RMI category (χ^2^(2) = 14.76, *p* < 0.001), whereas treatment group (χ^2^(1) = 0.19, *p* = 0.662), stroke stage (χ^2^(1) = 0.00, *p* = 0.997), and the overall Time × Treatment interaction (χ^2^(2) = 2.30, *p* = 0.316) were not statistically significant (Supplementary Figure S1; see Supplementary Table S3 for descriptive statistics for secondary outcomes at baseline, post-intervention, and follow-up, and within-group changes over time).

For concern about falling assessed with the FES-I, the collapsed ordinal model included 279 observations from 100 participants across baseline, post-intervention, and follow-up assessments and converged satisfactorily. In the RAGT+CPT reference group, the odds of being classified in a higher concern-about-falling category were significantly lower post-intervention than at baseline (OR = 0.001, 95% CI 0.00006–0.03; *p* < 0.001) and at follow-up than at baseline (OR = 0.002, 95% CI 0.0001–0.04; *p* < 0.001), indicating reduced concern about falling over time. At baseline, the CPT group did not differ significantly from the RAGT+CPT group (OR = 1.20, 95% CI 0.06–23.30; *p* = 0.905), and stroke stage was not significantly associated with FES-I category (OR = 0.46, 95% CI 0.03–8.19; *p* = 0.599). The Time × Treatment interactions were not statistically significant at either post-intervention (OR = 1.84, 95% CI 0.25–13.54; *p* = 0.548) or follow-up (OR = 1.43, 95% CI 0.20–10.28; *p* = 0.722), indicating no evidence that changes in concern about falling differed between treatment groups. Omnibus Wald tests confirmed a significant main effect of time on FES-I category (χ^2^(2) = 20.75, *p* < 0.001), whereas treatment group (χ^2^(1) = 0.12, *p* = 0.731), stroke stage (χ^2^(1) = 0.28, *p* = 0.599), and the overall Time × Treatment interaction (χ^2^(2) = 0.36, *p* = 0.835) were not statistically significant (Supplementary Figure S2).

For HRQoL assessed with the SF-SIS, the collapsed ordinal model included 279 observations from 100 participants across baseline, post-intervention, and follow-up assessments and converged satisfactorily. In the RAGT+CPT reference group, the odds of being classified in a higher functional status category were significantly higher post-intervention than at baseline (OR = 6.49, 95% CI 2.20–19.12; *p* < 0.001) and at follow-up than at baseline (OR = 10.42, 95% CI 3.25–33.39; *p* < 0.001), indicating improved self-reported functional status over time. At baseline, the CPT group did not differ significantly from the RAGT+CPT group (OR = 0.41, 95% CI 0.12–1.39; *p* = 0.153), and stroke stage was not significantly associated with SF-SIS category (OR = 0.93, 95% CI 0.34–2.52; *p* = 0.885). The Time × Treatment interactions were not statistically significant at either post-intervention (OR = 0.81, 95% CI 0.21–3.13; *p* = 0.764) or follow-up (OR = 0.45, 95% CI 0.11–1.86; *p* = 0.272), indicating no evidence that changes in SF-SIS category differed between treatment groups. Omnibus Wald tests confirmed a significant main effect of time on SF-SIS category (χ^2^(2) = 29.36, *p* < 0.001) and treatment group (χ^2^(1) = 5.16, *p* = 0.023), whereas stroke stage (χ^2^(1) = 0.02, *p* = 0.885) and the overall Time × Treatment interaction (χ^2^(2) = 1.25, *p* = 0.535) were not statistically significant (Supplementary Figure S3).

For EQ-5D-5L Index-assessed HRQoL, no significant time × intervention interaction (*p* = 0.957), time × stroke phase interaction (*p* = 0.657), or three-way time × intervention × stroke phase interaction (*p* = 0.177) was observed. A significant main effect of time was found, *F*(2, 84) = 28.46, *p* < 0.001, η^2^ₚ = 0.404, indicating improvements in HRQoL (EQ-5D-5L Index) over time across all participants.

For HRQoL assessed with the EQ-5D-5L VAS, the collapsed ordinal model included 279 observations from 100 participants across baseline, post-intervention, and follow-up assessments and converged satisfactorily. Higher categories represent better perceived health. In the RAGT+CPT reference group, the odds of being classified in a higher perceived-health category were significantly greater at post-intervention than at baseline (OR = 34.96, 95% CI 9.82 to 124.43, *p* < 0.001) and at follow-up than at baseline (OR = 35.11, 95% CI 9.89 to 124.72, *p* < 0.001). At baseline, the CPT group did not differ significantly from the RAGT+CPT group (OR = 1.63, 95% CI 0.36 to 7.35, *p* = 0.524), and stroke stage was not significantly associated with perceived-health category (OR = 0.68, 95% CI 0.19 to 2.49, *p* = 0.559). Significant Time × Treatment interactions were observed at both post-intervention (OR = 0.23, 95% CI 0.05 to 0.98, *p* = 0.047) and follow-up (OR = 0.05, 95% CI 0.01 to 0.23, *p* < 0.001), indicating that improvements in perceived health were significantly smaller in the CPT group than in the RAGT+CPT group at both time points. Omnibus Wald tests confirmed significant effects of Time (χ^2^(2) = 39.55, *p* < 0.001) and the Time × Treatment interaction (χ^2^(2) = 14.68, *p* = 0.001), whereas the main effects of treatment group (χ^2^(1) = 2.31, *p* = 0.129) and stroke stage (χ^2^(1) = 0.34, *p* = 0.559) were not statistically significant (Supplementary Figure S4).

For fatigue assessed with the FSS, the collapsed ordinal model included 278 observations from 100 participants across baseline, post-intervention, and follow-up assessments and converged satisfactorily. In the RAGT+CPT reference group, the odds of being classified in a higher (worse) fatigue category were significantly lower at post-intervention than at baseline (OR = 0.03, 95% CI 0.004 to 0.29, *p* = 0.002) and at follow-up than at baseline (OR = 0.06, 95% CI 0.008 to 0.41, *p* = 0.005). At baseline, the CPT group had significantly greater odds of being classified in a higher (worse) fatigue category than the RAGT+CPT group (OR = 13.60, 95% CI 1.33 to 139.27, *p* = 0.028), whereas stroke stage was not significantly associated with fatigue category (OR = 0.50, 95% CI 0.07 to 3.70, *p* = 0.494). A significant Time × Treatment interaction was observed at post-intervention (OR = 17.11, 95% CI 1.59 to 183.59, *p* = 0.019), indicating that improvements in fatigue were significantly smaller in the CPT group than in the RAGT+CPT group. The corresponding interaction at follow-up was not statistically significant (OR = 5.95, 95% CI 0.64 to 55.38, *p* = 0.117). Omnibus Wald tests confirmed significant effects of Time (χ^2^(2) = 13.60, *p* = 0.001) and treatment group (χ^2^(1) = 10.64, *p* = 0.001), whereas stroke stage (χ^2^(1) = 0.47, *p* = 0.494) and the Time × Treatment interaction (χ^2^(2) = 5.93, *p* = 0.051) were not statistically significant (Supplementary Figure S5). For details, see the Supplementary Table S3.

#### 3.4.3 Perceived exertion and intervention acceptability - weeks 1–4

Across the intervention period, perceived exertion was comparable between groups and remained within the moderate-to-strong range (median Borg CR10: 3.73; IQR 1.51–5.37), with no statistically significant between-group differences (*p* = 0.259; Supplementary Figure S6). Intervention acceptability was high across all groups, with median ratings of 1–2 on the 5-point Smiley Likert scale (lower scores indicating greater satisfaction), and no statistically significant between-group differences were observed for any acceptability item (Supplementary Figure S7).

#### 3.4.4 Training progression during robot-assisted gait training

Across successive training phases, participants in both RAGT+CPT groups demonstrated progressive increases in gait speed, walking distance, number of steps, and step length, indicating increasing training demands and walking capacity during the intervention. Training trajectories were comparable between groups.

## 4 Discussion

This study compared the effects of a time-matched 4-week program of RAGT combined with CPT versus CPT alone on walking ability, ADL, and related functional outcomes, and examined whether these effects differed between subacute and chronic stroke. Cumulative link mixed model analyses showed significant improvements over time in walking ability and basic ADL, with no evidence of significant Time × Treatment interactions or treatment-group effects for either primary outcome. Accordingly, *post hoc* analyses demonstrated significant improvements from baseline to post-intervention in both intervention groups, with no evidence that RAGT+CPT was superior to CPT alone. These findings indicate that intensive inpatient rehabilitation improved walking independence and basic ADL irrespective of treatment allocation and are consistent with previous evidence that functional recovery may continue beyond the early post-stroke period (70) although its extent likely varies according to baseline impairment, intervention intensity, and individual patient characteristics (71, 72).

Our findings differ from those of some previous studies reporting superiority of RAGT in subacute stroke (17) and from the Cochrane review by Mehrholz et al. (9) which concluded that electromechanical-assisted gait training combined with physiotherapy increases the likelihood of regaining independent walking, with the greatest benefits reported among participants treated during the earlier stages of stroke recovery. However, the same review also concluded that benefits in chronic stroke remain uncertain. Differences between studies may relate to variation in baseline severity, intervention intensity, participant selection, and comparator treatments. Despite randomization, some imbalance in baseline ambulatory function was observed between treatment groups, with a greater proportion of participants with severe walking impairment allocated to the RAGT+CPT group. Additional analyses adjusting for baseline FAC indicated that this baseline imbalance did not materially alter the principal intervention-related conclusions for the continuous walking outcomes. Participants in the present study were predominantly ambulatory at baseline, with relatively high FAC and Barthel Index scores. Specifically, 52% were independent walkers (FAC 4) and a further 23% were dependent walkers requiring supervision (FAC 3). The predominance of higher-functioning participants, together with the limited responsiveness of the FAC at the upper end of the scale, may have reduced the ability of this ordinal measure to detect additional between-group differences. Previous RAGT trials have frequently enrolled participants who were non-ambulatory or dependent walkers (FAC 0–3), particularly during the early subacute phase (<3 months) after stroke (9). Nevertheless, in the updated Cochrane review, almost half of all participants (49.7%; 4,224 participants overall) were independent walkers at baseline, and no significant subgroup differences according to baseline ambulatory status were identified (26). Accordingly, the predominantly ambulatory nature of our cohort may have reduced the opportunity to detect additional between-group differences in walking independence despite improvements observed in the more sensitive continuous walking outcomes.

For walking speed, no overall intervention × time interaction was observed. Although the primary analysis suggested greater improvement in participants with subacute than chronic stroke, this stroke phase effect was no longer statistically significant after adjustment for baseline ambulatory function, indicating that differences in baseline walking ability contributed to the apparent phase-related difference in recovery. Importantly, adjustment for baseline ambulatory function did not alter the intervention-related conclusions, and no evidence of superior effectiveness of RAGT+CPT over CPT was observed for walking speed. It should be noted that the definition of subacute stroke varies across studies; whereas Mehrholz et al. (9), defined the subacute phase as up to 3 months post-stroke, the present study followed the broader six-month definition proposed by Kwakkel et al. (35). Consequently, many participants classified as subacute in the present study were recruited beyond the earliest post-stroke recovery phase, during which previous studies have generally reported the greatest treatment effects of RAGT. This later timing of recruitment may partly explain why the present findings differ from studies that enrolled participants predominantly within the first 3 months after stroke.

The observed walking speed gains among subacute participants were broadly consistent with previous meta-analytic findings for end-effector RAGT (26, 73). By contrast, improvements in chronic-phase participants were smaller, suggesting that intervention responsiveness may decline over time. For walking distance, a significant three-way interaction indicated that endurance changes depended on both intervention type and stroke phase. Within the RAGT+CPT group, subacute participants achieved greater gains than chronic participants, whereas no phase-dependent difference was observed in the CPT group. This pattern remained significant after adjustment for baseline ambulatory function, supporting the robustness of the finding. Nevertheless, meaningful improvements in walking endurance were observed across the overall sample, supporting the effectiveness of intensive rehabilitation irrespective of stroke phase.

Falls decreased substantially over time across all groups and improvements were largely sustained at follow-up, although no between-group differences were identified. Functional mobility (TUG) also improved over time, with limited evidence for differential intervention effects. Similarly, functional mobility assessed with the RMI improved over time irrespective of treatment group, with no significant Time × Treatment interaction after adjustment for stroke stage. These findings are consistent with previous rehabilitation studies (28, 74) highlighting the importance of mobility recovery for safe participation in daily life (75). Overall, the objective functional outcomes showed comparable improvements following both interventions. HRQoL improved over time across interventions. While the SF-SIS and EQ-5D-5L Index showed no evidence of differential improvement between interventions, the EQ-5D-5L VAS demonstrated significantly greater improvements following RAGT+CPT than CPT after adjustment for stroke stage, although this finding was not consistently reflected across the other patient-reported outcomes. While objective functional outcomes were comparable between interventions, the greater improvement observed for the EQ-5D-5L VAS warrants further investigation to determine whether RAGT confers additional benefits for patient-perceived health. Similar improvements in quality of life following robotic rehabilitation have been reported previously (74, 76), although the mechanisms underlying these effects remain uncertain. Fatigue also improved over time, with evidence of a greater short-term reduction following RAGT+CPT than CPT, although this between-group difference was not maintained at follow-up and the overall Time × Treatment interaction narrowly missed statistical significance. These findings broadly align with previous robotic rehabilitation studies reporting improvements in fatigue without consistent evidence of sustained superiority over conventional rehabilitation (77).

Perceived exertion remained within a moderate-to-strong range throughout the intervention period, suggesting that both RAGT and CPT were delivered at a tolerable yet appropriately challenging intensity (78, 79). Device-recorded gait measures demonstrated improvements in gait speed, distance, step count, and step length across both subacute and chronic participants, indicating progressive adaptation to training over time. Intervention acceptability was high across all groups, with participants reporting strong satisfaction and no significant between-group differences in enjoyment or perceived effectiveness. These findings support the acceptability of both rehabilitation approaches within an inpatient setting and align with previous work emphasizing the importance of motivation, confidence, and therapeutic engagement during stroke rehabilitation (80–82). Consistent with our earlier feasibility work (13), factors such as clear communication, tactile support, and therapeutic rapport may have contributed positively to participant experience.

Several mechanisms may explain the functional improvements observed following both interventions. RAGT enables high-intensity, repetitive, task-specific gait training and provides multimodal sensory feedback, factors considered important for motor learning and neuroplastic adaptation (44, 83, 84). Neuroimaging studies have also reported increased motor cortical activity following robotic gait training (85). Conventional physiotherapy similarly incorporates task-specific repetitive practice, adaptive feedback, and strength and balance training, all of which are recognized components of post-stroke motor recovery (10, 86–88). However, these mechanisms were not directly assessed in the present study and should therefore be interpreted cautiously. Importantly, additional analyses adjusting for baseline ambulatory function did not materially alter the principal intervention-related conclusions, supporting the robustness of the findings despite the baseline imbalance in walking ability between treatment groups.

This study has several limitations. First, walking assessments could not be performed in participants who were non-ambulatory at baseline. Second, follow-up functional assessments were limited by discharge constraints, reducing interpretation of long-term effects. Third, because the intervention was delivered within a multidisciplinary inpatient rehabilitation program, the isolated contribution of RAGT cannot be fully determined. Fourth, strict eligibility criteria applied for safety during RAGT may limit generalisability. Fifth, although intention-to-treat analyses were performed, the relatively high and uneven attrition across groups may have reduced sensitivity to detect smaller effects. Sixth, some ordinal outcome categories were collapsed because of sparse observations, and three-way interaction models were simplified because of unstable parameter estimates. Consequently, several odds ratio estimates had wide confidence intervals, reflecting the modest sample size. In response to the observed baseline imbalance in ambulatory function, we also fitted cumulative link mixed models including baseline FAC as an additional covariate for the ordinal outcomes. However, these more highly parameterised models resulted in convergence warnings, a non-positive definite Hessian matrix, failure to estimate some parameters, and very wide confidence intervals, indicating that the models were overparameterised relative to the available data and did not provide sufficiently stable estimates for reliable inference. Accordingly, the prespecified cumulative link mixed models were retained for the ordinal outcomes, while the potential influence of baseline ambulatory function on the continuous outcomes was assessed using additional linear mixed-effects models adjusting for baseline FAC. In addition, the FAC may have limited responsiveness at the upper end of the scale, potentially reducing its ability to detect small but clinically meaningful improvements in higher-functioning participants. Furthermore, because the study population was predominantly ambulatory at baseline, the findings may not be generalisable to people with more severe walking impairments, who were underrepresented in the present cohort. Accordingly, the ordinal findings should be interpreted alongside the continuous outcome measures, which may be more sensitive to changes in walking performance and functional capacity. Seventh, neurophysiological and biomechanical mechanisms underlying functional improvements were not directly measured.

## 5 Conclusion

Among a predominantly ambulatory cohort of people with subacute and chronic stroke, RAGT combined with CPT did not demonstrate clear superiority over CPT alone for improving walking independence or other functional outcomes. Both interventions were associated with significant improvements over time in participants with subacute and chronic stroke, supporting the effectiveness of intensive inpatient rehabilitation beyond the early recovery phase. Improvements in walking endurance were more pronounced in subacute participants. Although the primary analyses suggested greater improvements in walking speed among participants with subacute stroke, this difference was attenuated after adjustment for baseline ambulatory function. Among the secondary outcomes, RAGT+CPT was associated with greater improvements in self-perceived health (EQ-5D-5L VAS), whereas evidence for a treatment advantage in fatigue was limited to the short term and most other outcomes were comparable between interventions. Overall, these findings suggest that RAGT may be most appropriately considered a complementary rehabilitation approach within multidisciplinary stroke rehabilitation rather than a universally superior alternative to conventional physiotherapy.

## Data Availability

The original contributions presented in the study are included in the article/Supplementary material, further inquiries can be directed to the corresponding author.

## Footnotes

1
https://drks.de/search/en/trial/DRKS00029910/details

## Glossary

ADL: Activities of daily living
ANOVA: Analysis of variance
BI: Barthel Index
Borg CR10: Borg Category Ratio scale
CI: Confidence interval
CONSORT: Consolidated Standards of Reporting Trials
CPT: Conventional physiotherapy
DRKS: German Clinical Trials Register
EQ-5D-5L: Euroqol 5-level EQ-5D
FAC: Functional Ambulation Categories
FES-I: Falls Efficacy Scale - International
FSS: Fatigue Severity Scale
HRQoL: Health-related quality of life
IQR: Interquartile range
MDR: Medical Device Regulation
MMST: Mini Mental Status Test
N: Number of participants
ProFaNE: Prevention of Falls Network Europe
r: Effect size (correlation coefficient)
RAGT: Robot-assisted gait training
RMI: Rivermead Mobility Index
SF-SIS: Short Form Stroke Impact Scale
TUG: Timed Up and Go
VAS: Visual Analogue Scale
Wk: Week
6MWT: 6-Meter Walk Test
10MWT: 10-Meter Walk Test
η^2^: Eta-squared
