# Supplementary material for: Robot-assisted gait training in people with subacute and chronic stroke: a randomized controlled trial
Source: Front Neurol. 2026 Sep 3;17:1898475. doi: 10.3389/fneur.2026.1898475 (PMC13581499; doi:10.3389/fneur.2026.1898475)
Supplement: Supplementary file 1 [file Data_Sheet_1.PDF]

Supplementary Material

File S1. Comprehensive list of exclusion criteria.

Exclusion criteria were: comorbidities such as malignant diseases, severe neurological, orthopaedic, or psychiatric conditions, cardiac contraindications; acute severe pain despite conventional pain management; joint contractures and arthrodesis; significant reduction in bone density with an increased risk of fractures; bone or joint instability; severe vascular diseases of the lower extremities or skin lesions at sites in contact with the device or harness system; severe spasticity in the lower extremities; or significantly disproportionate growth of the legs and/or spine. Exclusion also applied to people outside body weight (15-180 kg) or height (100-200 cm) limits; with a health condition preventing active rehabilitation; severe apraxia or severe osteoarthritis in the joints of the lower extremities; uncooperative or self-aggressive behaviour, cardiac conditions including heart failure, thoracotomy, uncontrolled orthostatic hypotension, or other circulatory problems; impaired blood flow in the lower extremities; recent joint injuries or endoprostheses with contraindicated ranges of motion or loading limits; consolidated fractures in the lower extremities within six months post-injury; epilepsy, recent seizures, or an elevated seizure risk; enterostomy; stoma; sensory disturbances in the lower extremities or trunk; or pregnancy. Recent medication changes affecting gait or functional performance also necessitated exclusion.

File S2. Detailed description of assessments used in this study.

The 10MWT is a performance measure used to assess walking speed in metres per second over a short distance. It can evaluate functional mobility across various neurological populations (1). The setup requires a stopwatch and a clear 10-metre walkway, marked at 0, 2, 8, and 10 metres. The participant walks unaided for 10 metres, with timing recorded for the middle 6 metres to account for acceleration and deceleration. Assistive devices may be used but must be consistent and documented. The test administrator should walk at least half a step behind to avoid influencing the pace. Timing starts when the toes pass the 2-metre mark and stops at the 8-metre mark. Two trials are conducted at the participant's maximum speed, with the results averaged and recorded in metres/second (2).

The 6MWT measures the distance a person can walk in six minutes and is commonly used to assess individuals with stroke (3). Participants completed the test along a 30-metre corridor, aiming to cover as much ground as possible with or without assistive devices and without physical assistance. No warm-up is permitted beforehand, following American Thoracic Society guidelines (4). After resting for at least 10 minutes near the start, they walk back and forth, with rests possible at any time - recorded by number and duration. Assistive devices are allowed but must be consistent and documented. Participants turn at each end and are guided by standard verbal encouragement every minute. The total laps and final partial lap distance are recorded, with distance measured using a calibrated wheel (4).

The TUG test evaluates functional mobility. The patient sits upright in a chair; on the command 'go,' they stand, walk 3 metres at a comfortable pace, turn, return to the chair, and sit down. Timing begins at 'go' and stops when the patient is seated. The time in seconds, from the command to

start until the buttocks touch the chair, is recorded. One practice trial is recommended but not included in the score. The same assistive device must be used for each test, and no physical assistance is provided (5).

The RMI was developed from the Rivermead Motor Assessment Gross Function subscale to quantify functional mobility disability in individuals after stroke (6). The validated German version was used in this study (7). It includes fifteen mobility items: 14 self-reported and 1 direct observation (standing unsupported). The items are hierarchically arranged according to Guttmann scaling criteria, indicating they are ordered by increasing difficulty (8). Success in more difficult items suggests the client is likely to succeed in easier ones. Each item is scored as 0 or 1, with 0 indicating a 'no' response and 1 a 'yes', and the total score is obtained by summing all points. The maximum score is 15, with higher scores reflecting better mobility (6).

The validated German version of the 16-item FES-I was used to assess concerns about falling in study participants (9). Developed through a series of meetings with the Prevention of Falls Network Europe (ProFaNE), a European Commission-funded collaboration, it is a patient-reported outcome measure covering various functional activities. Participants rate each activity on a four-point Likert scale, indicating their level of concern about falling if they performed the activity, regardless of whether they actually perform it (10, 11). Scores range from 1 (not at all concerned) to 4 (very concerned) for each item, with total scores summing from 16 to 64. Higher scores signify greater concern about falling (10).

The validated German version of the 8-item SIS-SF was used in this study to assess HRQoL (12). The SF-SIS (13, 14) is a shortened version of the Stroke Impact Scale 2.0 (15), designed to reduce patient burden while providing a reliable measure of disability and HRQoL post-stroke. It selects one item from each of the eight SIS 2.0 domains - strength, hand function, ADL / instrumental ADL, mobility, communication, emotion, memory, and participation - that best reflect each domain's core concept (15). Ratings are based on a 5-point Likert scale from 1 ('could not do at all') to 5 ('not difficult at all'). The total score, ranging from 8 to 40, is converted into an interval-scaled index from 0 to 100 points, with higher scores indicating better HRQoL (12).

The validated German version of EQ-5D-5L (16) was used to assess HRQoL in this study. The EQ-5D-5L consists of two parts: the descriptive system and the visual analogue scale (VAS). The descriptive system includes five dimensions - mobility, self-care, usual activities, pain/discomfort, and anxiety/depression - each with five levels from 'no problems' to 'extreme problems.' Individuals select the statement that best describes their health in each dimension. Using an SPSS syntax from the Euroqol website (https://euroqol.org/information-and-support/resources/value-sets/), these responses can be translated into one of 3,125 health states, from 'no problems' (level 1) to 'extreme problems' (level 5) or expressed as a single index value (EQ Index) ranging from below 0 to 1, with higher scores indicating better HRQoL. The EQ VAS records the patient's self-rated health on a scale from 0 ('worst health you can imagine') to 100 ('best health you can imagine'), where the patient marks their current health and clarifies the score with a an 'X' and a corresponding number (17).

The validated German version of the 9-item FSS was used to assess fatigue in this study (18). The FSS is widely used to measure fatigue in individuals with chronic illnesses, including stroke (19). Each item is scored on a seven-point Likert scale from 1 (‘strongly disagree’) to 7 (‘strongly agree’). Scores range from 9 to 63, with higher scores indicating greater fatigue severity (20).

A 5-point Smiley Likert Scale was used to measure intervention acceptability, with lower scores indicating higher satisfaction (21). Four questions were asked: ‘How did you like the training?” (1); ‘How did you feel during the training?” (2); ‘How satisfied were you with your performance?” (3); and ‘How satisfied were you with your results?” (4).

As additional secondary outcomes, gait parameters (speed, distance, step count, and step length) were recorded by the robotic gait trainer during RAGT sessions only, in participants in the subacute and chronic phases post stroke. The first session was considered an introductory session and was excluded from analysis. Sessions 2–5, 6–8, 9–12, and 13–16 were clustered into four distinct time periods for analysis. Comparisons were conducted within and between groups following the intervention to evaluate changes across the intervention period.

References

1. Flansbjer UB, Holmbäck AM, Downham D, Patten C, Lexell J. Reliability of gait performance tests in men and women with hemiparesis after stroke. J Rehabil Med. 2005;37(2):75-82.
2. Bohannon RW. Comfortable and maximum walking speed of adults aged 20-79 years: reference values and determinants. Age Ageing. 1997;26(1):15-9.
3. Kosak M, Smith T. Comparison of the 2-, 6-, and 12-minute walk tests in patients with stroke. J Rehabil Res Dev. 2005;42(1):103-7.
4. Laboratories ATSCoPSfCPF. ATS statement: guidelines for the six-minute walk test. Am J Respir Crit Care Med. 2002;166(1):111-7.
5. Podsiadlo D, Richardson S. The timed "Up & Go": a test of basic functional mobility for frail elderly persons. J Am Geriatr Soc. 1991;39(2):142-8.
6. Collen FM, Wade DT, Robb GF, Bradshaw CM. The Rivermead Mobility Index: a further development of the Rivermead Motor Assessment. Int Disabil Stud. 1991;13(2):50-4.
7. Schindl MR, Forstner C, Kern H, Zipko HT, Rupp M, Zifko UA. Evaluation of a German version of the Rivermead Mobility Index (RMI) in acute and chronic stroke patients. Eur J Neurol. 2000;7(5):523-8.
8. Roorda LD, Green JR, Houwink A, Bagley PJ, Smith J, Molenaar IW, et al. Item hierarchy-based analysis of the Rivermead Mobility Index resulted in improved interpretation and enabled faster scoring in patients undergoing rehabilitation after stroke. Arch Phys Med Rehabil. 2012;93(6):1091-6.
9. Dias N, Kempen GI, Todd CJ, Beyer N, Freiberger E, Piot-Ziegler C, et al. [The German version of the Falls Efficacy Scale-International Version (FES-I)]. Z Gerontol Geriatr. 2006;39(4):297-300.
10. Dewan N, MacDermid JC. Fall Efficacy Scale - International (FES-I). Journal of Physiotherapy. 2014;60(1):60.

11. Yardley L, Beyer N, Hauer K, Kempen G, Piot-Ziegler C, Todd C. Development and initial validation of the Falls Efficacy Scale-International (FES-I). Age Ageing. 2005;34(6):614-9.
12. Coppers A, Moller JC, Marks D. Psychometric properties of the short form of the Stroke Impact Scale in German-speaking stroke survivors. Health Qual Life Outcomes. 2021;19(1):190.
13. Jenkinson C, Fitzpatrick R, Crocker H, Peters M. The Stroke Impact Scale: validation in a UK setting and development of a SIS short form and SIS index. Stroke. 2013;44(9):2532-5.
14. MacIsaac R, Ali M, Peters M, English C, Rodgers H, Jenkinson C, et al. Derivation and Validation of a Modified Short Form of the Stroke Impact Scale. J Am Heart Assoc. 2016;5(5).
15. Petersen C, Morfeld M, Bullinger M. [Testing and validation of the German version of the Stroke Impact Scale]. Fortschr Neurol Psychiatr. 2001;69(6):284-90.
16. Ludwig K, von der Schulenburg JG, Greiner W. Valuation of the EQ-5D-5L with composite time trade-off for the German population - an exploratory study. Health Qual Life Outcomes. 2017;15(1):39.
17. Herdman M, Gudex C, Lloyd A, Janssen M, Kind P, Parkin D, et al. Development and preliminary testing of the new five-level version of EQ-5D (EQ-5D-5L). Quality of life research : an international journal of quality of life aspects of treatment, care and rehabilitation. 2011;20(10):1727-36.
18. Valko PO, Bassetti CL, Bloch KE, Held U, Baumann CR. Validation of the fatigue severity scale in a Swiss cohort. Sleep. 2008;31(11):1601-7.
19. Taghizadeh G, Sarlak N, Fallah S, Sharabiani PTA, Cheraghifard M. Minimal clinically important differenceof fatigue severity scale in patients with chronic stroke. J Stroke Cerebrovasc Dis. 2024;33(4):107577.
20. Krupp LB, LaRocca NG, Muir-Nash J, Steinberg AD. The fatigue severity scale. Application to patients with multiple sclerosis and systemic lupus erythematosus. Arch Neurol. 1989;46(10):1121-3.
21. Uestuener P, Ferrarini A, Santi M, Mardegan C, Bianchetti MG, Simonetti GD, et al. Taste acceptability of pulverized brand-name and generic drugs containing amlodipine or candesartan. Int J Pharm. 2014;468(1-2):196-8.

Table S1. Adverse events, relatedness, and event categories by study group.

**Variable**	**G1: RAGT+CPT subacute stroke (n=25)**	**G2: CPT subacute stroke (n=25)**	**G3: RAGT+CPT chronic stroke (n=25)**	**G4: CPT chronic stroke (n=25)**	**Total (n=100)**
Participants with ≥1 AE	9	8	7	7	31
Total AEs	10	9	8	7	34
SAEs	1*	0	0	0	1
Possibly related AEs	2	0	1	0	3
AE-associated discontinuations	7	1	1	1	10

**Adverse event type**	**Groups affected**	**Total**
Muscle soreness	All groups	10
Fatigue	All groups	9
Musculoskeletal pain	All groups	5
COVID-19 / respiratory tract infection	G1, G2, G4	4
Headache	G2, G4	2
Pain after fall	G1	2
Knee pain associated with incipient gonarthrosis	G3	1

Data are presented as number of participants. Adverse events were classified according to ISO 14155 definitions. Relationship to the intervention was assessed by the investigators.

* One fall-related femoral neck fracture judged unrelated to the intervention.

AE, adverse event; SAE, serious adverse event; RAGT, robot-assisted gait training; CPT, conventional physiotherapy.

Table S2. Descriptive statistics for the primary outcomes at baseline and post-intervention, and within-group changes over time.

Parameter	RAGT+CPT subacute stroke (n=25)	CPT subacute stroke (n=25)	RAGT+CPT chronic stroke (n=25)	CPT chronic stroke (n=25)
BL FAC[a]	4 (0-4)	4 (1-4)	3 (0-4)	3 (1-4)
PI FAC[a]	5 (1-5)	4 (2-5)	4 (1-5)	4 (1-5)
Δ PI-BL FAC	1 (0-3)	0.5 (0-2)	1 (0-2)	1 (0-2)
BL BI[a]	90 (15-100)	85 (35-100)	80 (25-100)	80 (40-100)
PI BI[a]	100 (25-100)	90 (40-100)	93 (25-100)	95 (45-100)
Δ PI-BL BI	10 (0 to 65)	5 (-5 to 25)	5 (0 to 55)	5 (-5 to 35)

Data represent median (range). Higher FAC and BI values indicate better function. Abbreviations: BI, Barthel Index; BL, baseline; CPT, conventional physiotherapy; FAC, Functional Ambulation Categories; Δ (Delta), difference; PI, post-intervention; RAGT, robot-assisted gait training.

Table S3. Descriptive statistics for secondary outcomes assessing falls, functional mobility, concerns about falling, health-related quality of life, and fatigue at baseline, post-intervention, and follow-up, and within-group changes over time.

**Parameter**	**RAGT+CPT subacute stroke (n=25)**	**CPT subacute stroke (n=25)**	**RAGT+CPT chronic stroke (n=25)**	**CPT chronic stroke (n=25)**
BL falls (n)	8	14	5	8
BL fallers (n, %)	5 (5.0)	7 (7.0)	4 (4.0)	5 (5.0)
BL nonfallers (n, %)	20 (20.0)	18 (18.0)	21 (21.0)	20 (20.0)
PI falls (n)	1	1	2	3
PI fallers (n, %)	1 (1.1)	1 (1.1)	2 (2.2)	3 (3.3)
PI nonfallers (n, %)	17 (18.9)	23 (25.6)	22 (24.4)	21 (23.3)
FU falls (n)	0	4	2	1
FU fallers (n, %)	0 (0.0)	3 (3.3)	2 (2.2)	1 (1.1)
FU nonfallers (n, %)	18 (19.8)	21 (23.1)	22 (24.2)	24 (26.4)
BL RMI[a]	11 (2-15)	11 (4-15)	12 (2-14)	11 (6-14)
PI RMI[a]	14 (4-15)	12 (4-15)	13 (4-15)	12 (6-15)
FU RMI[a]	14 (5-15)	13 (6-15)	13 (5-15)	12 (7-15)
Δ PI-BL RMI[a]	1.5 (0 to 5)	1 (-3 to 5)	0 (-2 to 3)	0 (-5 to 3)
Δ FU-BL RMI[a]	1.5 (0 to 7)	2 (-2 to 5)	0.5 (-3 to 5)	1 (-3 to 4)
Δ FU-PI RMI[a]	0 (-1 to 4)	0 (-5 to 3)	-0.5 (-3 to 2)	0 (-1 to 5)
BL FES-I[a]	24 (16-64)	28 (18-58)	24 (17-60)	23 (16-43)
PI FES-I[a]	18 (16-39)	21 (16-55)	20 (16-53)	18 (16-34)
FU FES-I[a]	19 (16-38)	22 (16-43)	19 (16-51)	19 (16-36)
Δ PI-BL FES-I[a]	-4.5 (-34 to 0)	0-6 (-28 to -6)	-4 (-26 to 0)	-4 (-17 to 0)
Δ FU-BL FES-I[a]	-3.5 (-34 to 15)	-6.5 (-28 to -1)	-4.5 (-28 to 0)	-4 (-16 to 0)
Δ FU-PI FES-I[a]	0 (-2 to 17)	0 (-12 to 5)	0 (-5 to 3)	0 (-2 to 4)
BL SF-SIS[a]	78.13 (18.75-96.88)	65.63 (31.25-100.00)	68.75 (28.13-93.75)	68.75 (40.63-90.63)
PI SF-SIS[a]	85.94 (62.50-100)	79.69 (20-96.88)	85.94 (53.13-96.88)	75.00 (53.13-100)
FU SF-SIS[a]	84.37 (37.50-100)	81.25 (37.50-100)	81.25 (53.12-96.87)	75.00 (31.25-100)
Δ PI-BL SF-SIS[a]	12.5 (-3.13 to 37.5)	9.38 (-46.88 to 56.25)	9.38 (-3.13 to 31.25)	10.94 (-31.25 to 21.88)

Δ FU-BL SF-SIS[a]	10.94 (-12.50 to 46.88)	15.63 (-21.88 to 50.00)	7.81 (-6.25 to 31.25)	3.13 (-21.88 to 43.75)
Δ FU-PI SF-SIS[a]	1.56 (-37.5 to 15.63)	3.13 (-43.75 to 71.88)	-3.13 (-12.50 to 21.88)	-3.13 (-28.13 to 46.88)
BL SF- EQ-5D-5L Index[b]	0.75 ± 0.26	0.73 ± 0.24	0.66 ± 0.30	0.76 ± 0.20
PI SF- EQ-5D-5L Index[b]	0.85 ± 0.19	0.88 ± 0.12	0.86 ± 0.15	0.88 ± 0.13
FU SF- EQ-5D-5L Index[b]	0.85 ± 0.20	0.85 ± 0.13	0.81 ± 0.23	0.86 ± 0.15
Δ PI-BL EQ-5D-5L Index[b]	0.09 ± 0.13	0.17 ± 0.17	0.19 ± 0.25	0.13 ± 0.16
Δ FU-BL EQ-5D-5L Index[b]	0.09 ± 0.18	0.13 ± 0.19	0.14 ± 0.28	0.11 ± 0.18
Δ FU-PI EQ-5D-5L Index[b]	0 ± 0.14	-0.04 ± 0.08	-0.05 ± 0.16	-0.02 ± 0.10
BL SF- EQ-5D-5L VAS[a]	60 (25-85)	60 (20-80)	50 (15-85)	65 (25-100)
PI SF- EQ-5D-5L VAS[a]	80 (50-90)	70 (40-100)	70 (30-99)	70 (20-100)
FU SF- EQ-5D-5L VAS[a]	78 (50-90)	60 (50-90)	70 (40-95)	60 (40-80)
Δ PI-BL EQ-5D-5L VAS[a]	15 (5 to 30)	10 (-10 to 40)	10 (0 to 40)	2.5 (-13 to 25)
Δ FU-BL EQ-5D-5L VAS[a]	10 (0 to 45)	5 (-20 to 40)	10 (0 to 45)	0 (-30 to 15)
Δ FU-PI EQ-5D-5L VAS[a]	0 (-10 to 20)	-5 (-30 to 30)	0 (-5 to 20)	-5 (-30 to 20)
BL FSS[a]	30 (11-59)	34 (12-63)	28 (9-63)	33 (9-62)
PI FSS[a]	22 (10-38)	33 (10-59)	19 (9-63)	32 (12-60)
FU FSS[a]	20 (9-49)	31.5 (9-55)	18 (9-37)	33 (11-57)
Δ PI-BL FSS[a]	-6.5 (-19 to 8)	0 (-32 to 10)	-2.5 (-27 to 6)	-2.5 (-36 to 23)
Δ FU-BL FSS[a]	-6 (-24 to 12)	-4 (-31 to 10)	-7 (-29 to 5)	-2 (-33 to 18)
Δ FU-PI FSS[a]	-1 (-11 to 14)	-2 (-14 to 17)	-1 (-8 to 2)	-1 (-3 to 12)

[a]Median (range); [b]Mean (standard deviation). Higher RMI, SF-SIS, and EQ-5D-5L Index and VAS values indicate better function or health; lower FES-I and FSS values indicate better function. Abbreviations: BL, baseline; CPT, conventional physiotherapy; Δ (Delta), difference; EQ-5D-5L, EuroQol; FES-I, Falls Efficacy Scale-International; FSS, Fatigue Severity Scale; PI, post-intervention; RAGT, robot-assisted gait training; RMI, Rivermead Mobility Index; SF-SIS, Short Form Stroke Impact Scale; VAS, Visual Analogue Scale.

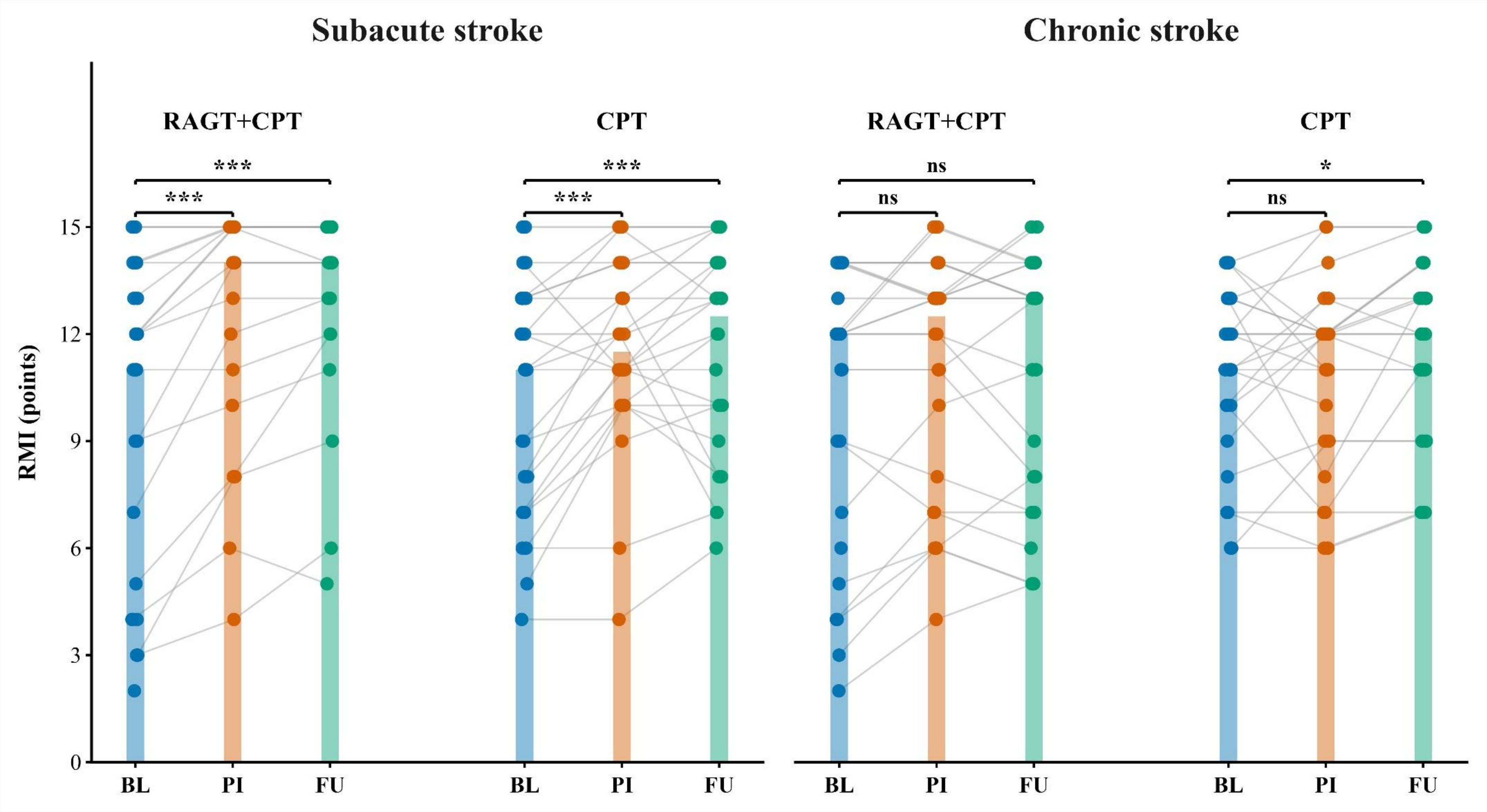

Figure S1. Changes in Rivermead Mobility Index (RMI) scores from baseline to post-intervention and follow-up by treatment group and stroke stage.

Rivermead Mobility Index (RMI) scores are shown for participants with subacute and chronic stroke, separately for RAGT+CPT and CPT. In the paired plots, blue, orange, and green points represent baseline (BL), post-intervention (PI), and follow-up (FU), respectively. Individual points represent participant-level observations, and grey lines connect repeated measurements within participants. Statistical analyses were performed using cumulative link mixed models (CLMMs) based on collapsed FES-I categories, with fixed effects for time, treatment group, the Time × Treatment interaction, and stroke stage, and a participant-specific random intercept. Brackets indicate model-based within-group comparisons between time points within each treatment group and stroke-stage stratum. Lower FES-I scores indicate less concern about falling. Significance levels are indicated as ns, not significant; *$p < 0.05$; **$p < 0.01$; and ***$p < 0.001$. Abbreviations: FES-I, Falls Efficacy Scale–International; BL, baseline; PI, post-intervention; FU, follow-up; RAGT+CPT, robot-assisted gait training plus conventional physiotherapy; CPT, conventional physiotherapy.

Alt text: Paired plot showing Rivermead Mobility Index (RMI) scores at baseline (blue), post-intervention (orange), and follow-up (green) for participants with subacute and chronic stroke in the RAGT+CPT and CPT groups. Individual participant observations are connected by grey lines to indicate repeated measurements. Brackets indicate model-based within-group comparisons with significance annotations.

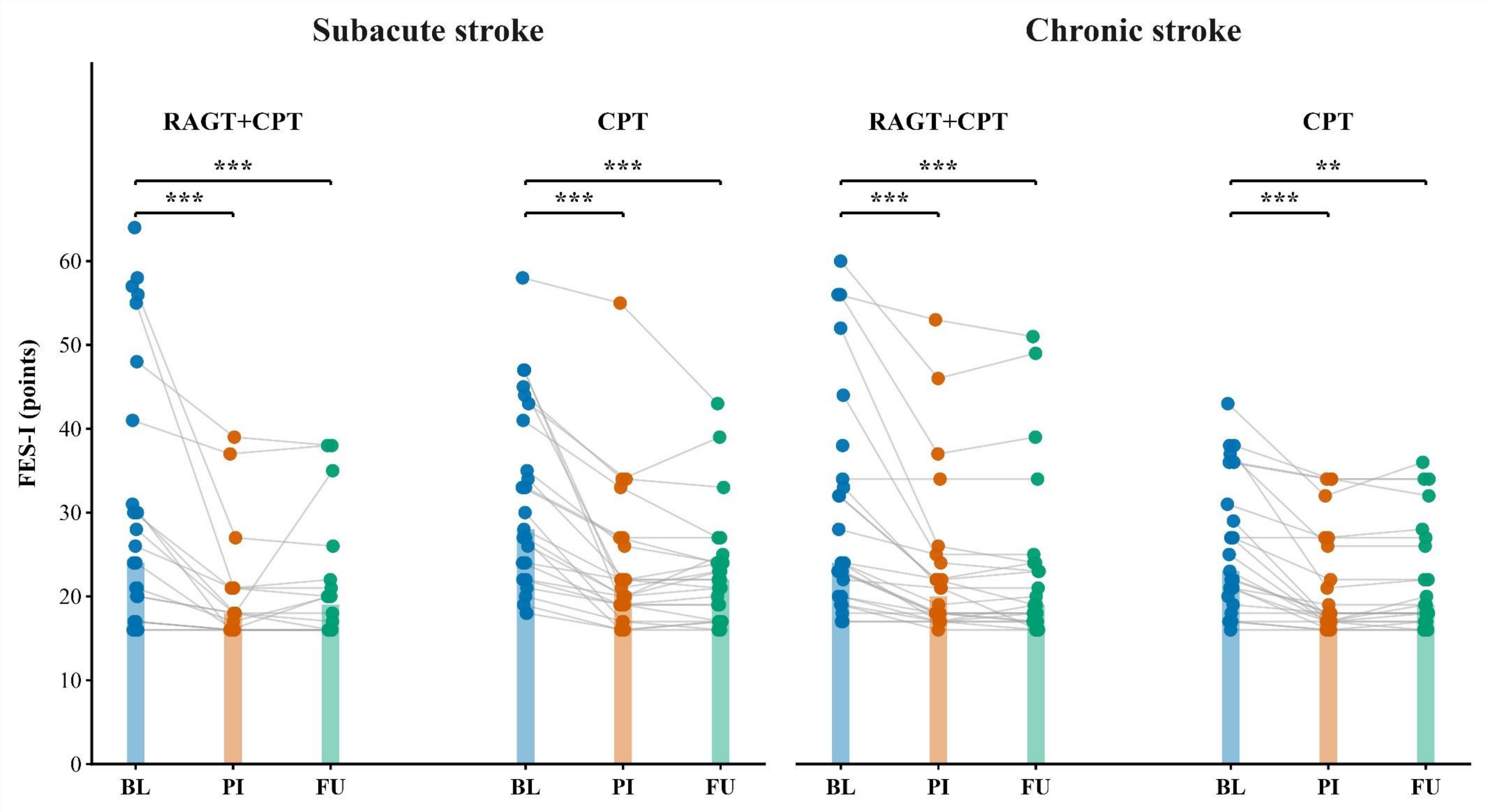

Figure S2. Changes in Falls Efficacy Scale–International (FES-I) scores from baseline to post-intervention and follow-up by treatment group and stroke stage.

Falls Efficacy Scale–International (FES-I) scores are shown for participants with subacute and chronic stroke, separately for RAGT+CPT and CPT. Blue, orange, and green paired data points represent baseline (BL), post-intervention (PI), and follow-up (FU), respectively. Individual points represent participant-level observations, and grey lines connect repeated measurements within participants. Statistical analyses were performed using cumulative link mixed models (CLMMs) based on collapsed FES-I categories, with fixed effects for time, treatment group, the Time × Treatment interaction, and stroke stage, and a participant-specific random intercept. Brackets indicate model-based within-group comparisons between time points within each treatment group and stroke-stage stratum. Lower FES-I scores indicate less concern about falling. Significance levels are indicated as ns, not significant; *$p < 0.05$; **$p < 0.01$; and ***$p < 0.001$. Abbreviations: FES-I, Falls Efficacy Scale–International; BL, baseline; PI, post-intervention; FU, follow-up; RAGT+CPT, robot-assisted gait training plus conventional physiotherapy; CPT, conventional physiotherapy.

Alt text: Paired plot showing Falls Efficacy Scale–International (FES-I) scores at baseline (blue), post-intervention (orange), and follow-up (green) for participants with subacute and chronic stroke in the RAGT+CPT and CPT groups. Individual participant observations are connected by grey lines to indicate repeated measurements. Brackets indicate model-based within-group comparisons with significance annotations.

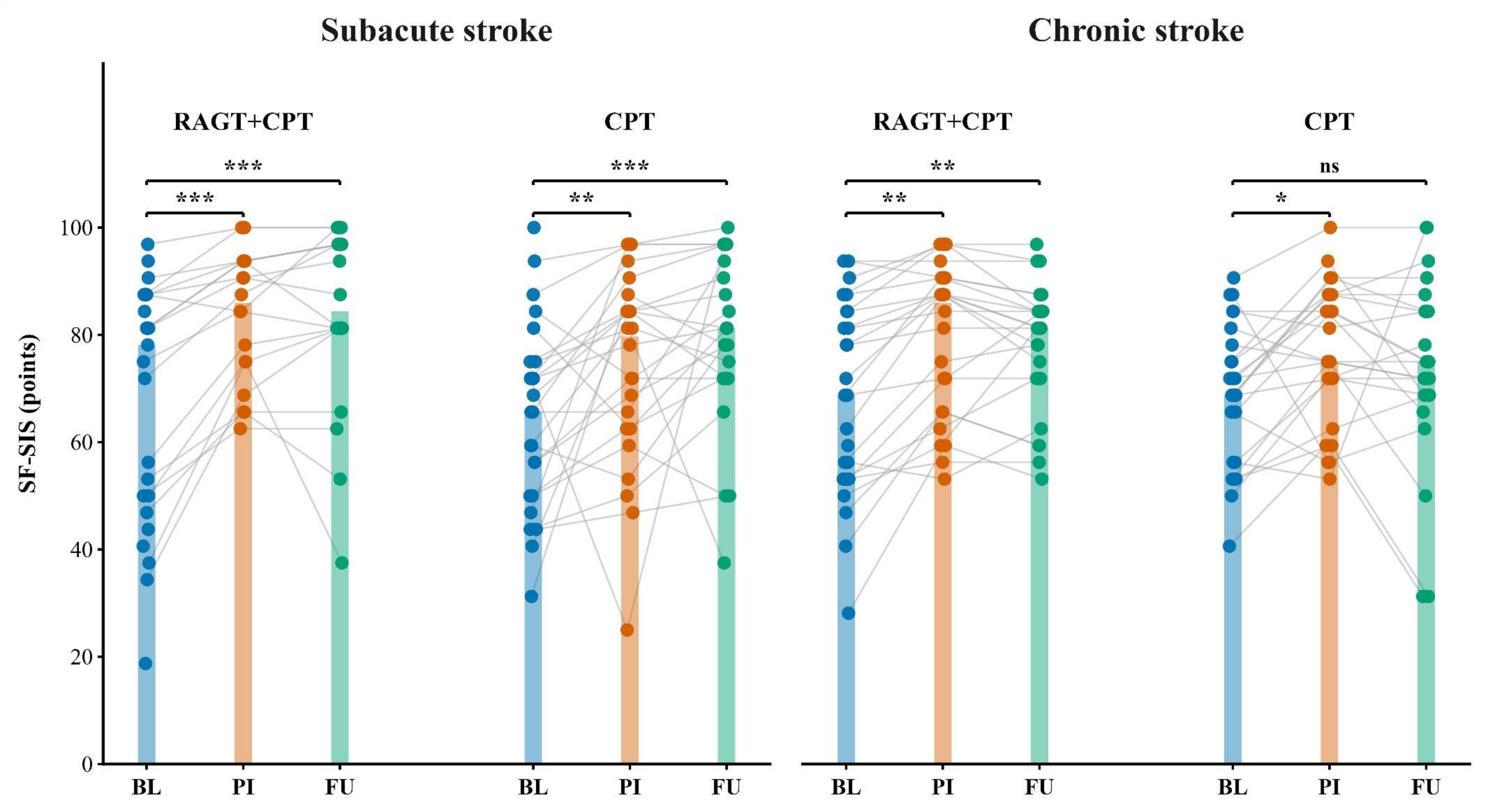

Figure S3. Changes in Short Form Stroke Impact Scale (SF-SIS) scores from baseline to post-intervention and follow-up by treatment group and stroke stage.

Short Form Stroke Impact Scale (SF-SIS) scores are shown for participants with subacute and chronic stroke, separately for RAGT+CPT and CPT. In the paired plots, blue, orange, and green points represent baseline (BL), post-intervention (PI), and follow-up (FU), respectively. Individual points represent participant-level observations, and grey lines connect repeated measurements within participants. Statistical analyses were performed using cumulative link mixed models (CLMMs) based on collapsed SF-SIS categories, with fixed effects for time, treatment group, the Time × Treatment interaction, and stroke stage, and a participant-specific random intercept. Brackets indicate model-based within-group comparisons between time points within each treatment group and stroke-stage stratum. Higher SF-SIS scores indicate better self-reported functional status and health-related quality of life. Significance levels are indicated as ns, not significant; $^*p < 0.05$; $^{**}p < 0.01$; and $^{***}p < 0.001$. Abbreviations: SF-SIS, Short Form Stroke Impact Scale; BL, baseline; PI, post-intervention; FU, follow-up; RAGT+CPT, robot-assisted gait training plus conventional physiotherapy; CPT, conventional physiotherapy.

Alt text: Paired plot showing Short Form Stroke Impact Scale (SF-SIS) scores at baseline (blue), post-intervention (orange), and follow-up (green) for participants with subacute and chronic stroke in the RAGT+CPT and CPT groups. Individual participant observations are connected by grey lines to indicate repeated measurements. Brackets indicate model-based within-group comparisons with significance annotations.

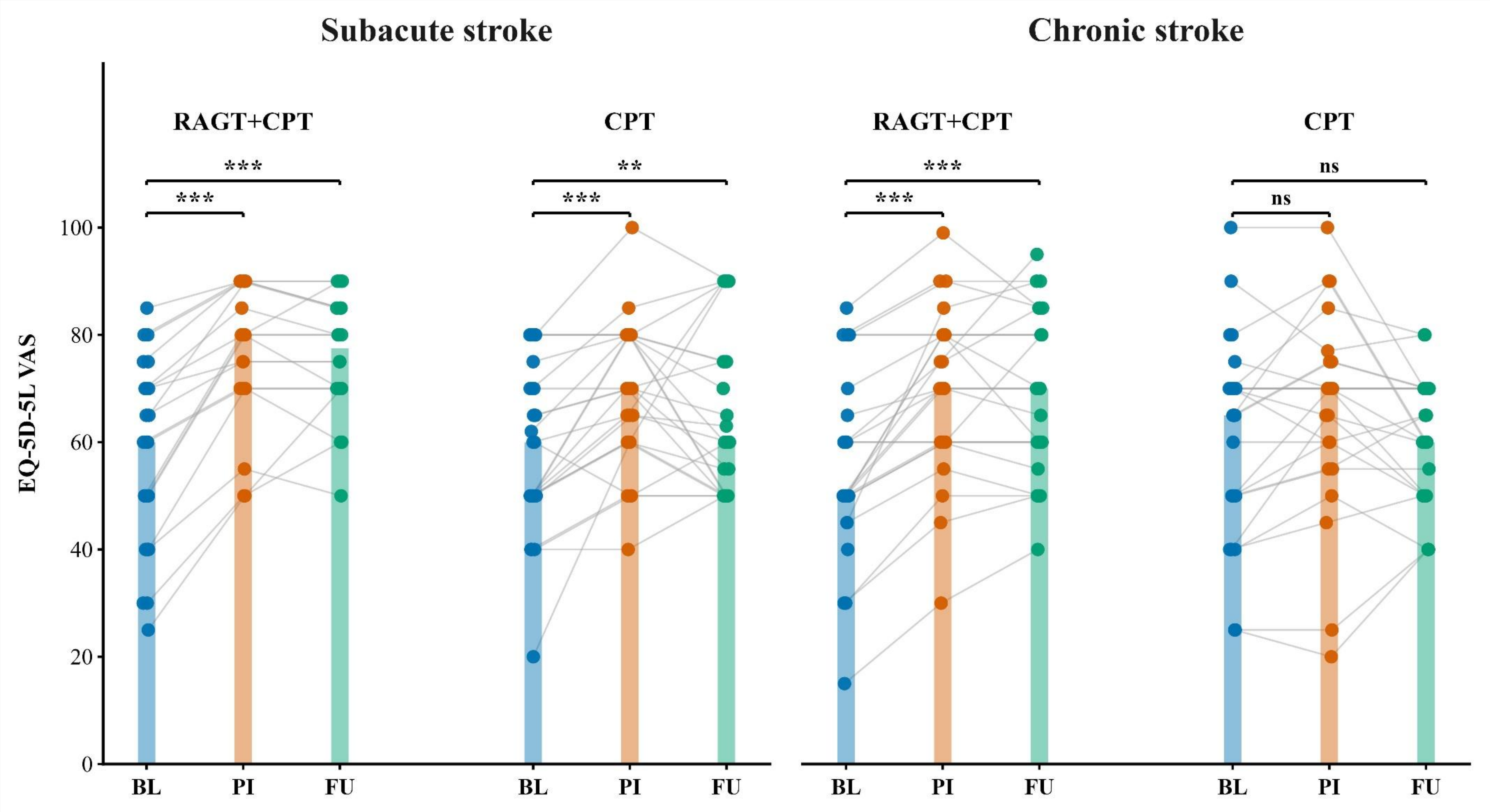

Figure S4. Changes in EuroQol 5-Dimension 5-Level Visual Analogue Scale (EQ-5D-5L VAS) scores from baseline to post-intervention and follow-up by treatment group and stroke stage.

EQ-5D-5L VAS scores are shown for participants with subacute and chronic stroke, separately for RAGT+CPT and CPT. In the paired plots, blue, orange, and green points represent baseline (BL), post-intervention (PI), and follow-up (FU), respectively. Individual points represent participant-level observations, and grey lines connect repeated measurements within participants. Statistical analyses were performed using cumulative link mixed models (CLMMs) based on collapsed EQ-5D-5L VAS categories, with fixed effects for time, treatment group, the Time × Treatment interaction, and stroke stage, and a participant-specific random intercept. Brackets indicate model-based within-group comparisons between time points within each treatment group and stroke-stage stratum. Higher EQ-5D-5L VAS scores indicate better self-reported health status. Significance levels are indicated as ns, not significant; $*p < 0.05$; $**p < 0.01$; and $***p < 0.001$. Abbreviations: EQ-5D-5L VAS, EuroQol 5-Dimension 5-Level Visual Analogue Scale; BL, baseline; PI, post-intervention; FU, follow-up; RAGT+CPT, robot-assisted gait training plus conventional physiotherapy; CPT, conventional physiotherapy.

Alt text: Paired plot showing EQ-5D-5L Visual Analogue Scale (EQ-5D-5L VAS) scores at baseline (blue), post-intervention (orange), and follow-up (green) for participants with subacute and chronic stroke in the RAGT+CPT and CPT groups. Individual participant observations are connected by grey lines to indicate repeated measurements. Brackets indicate model-based within-group comparisons with significance annotations.

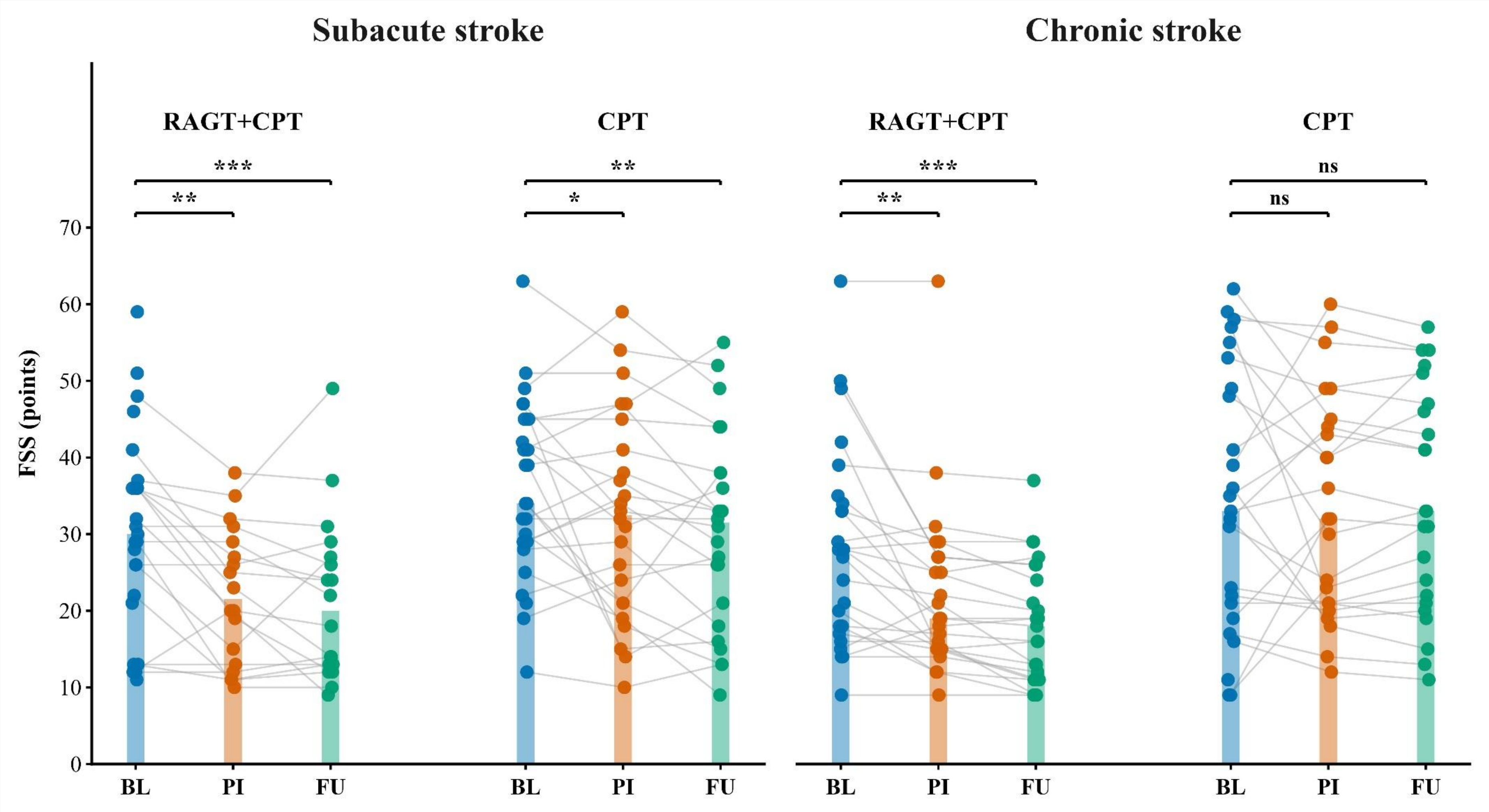

Figure S5. Changes in Fatigue Severity Scale (FSS) scores from baseline to post-intervention and follow-up by treatment group and stroke stage.

Fatigue Severity Scale (FSS) scores are shown for participants with subacute and chronic stroke, separately for RAGT+CPT and CPT. In the paired plots, blue, orange, and green points represent baseline (BL), post-intervention (PI), and follow-up (FU), respectively. Individual points

represent participant-level observations, and grey lines connect repeated measurements within participants. Statistical analyses were performed using cumulative link mixed models (CLMMs) based on collapsed FSS categories, with fixed effects for time, treatment group, the Time × Treatment interaction, and stroke stage, and a participant-specific random intercept. Brackets indicate model-based within-group comparisons between time points within each treatment group and stroke-stage stratum. Lower FSS scores indicate less fatigue. Significance levels are indicated as ns, not significant; *$p < 0.05$; **$p < 0.01$; and ***$p < 0.001$. Abbreviations: FSS, Fatigue Severity Scale; BL, baseline; PI, post-intervention; FU, follow-up; RAGT+CPT, robot-assisted gait training plus conventional physiotherapy; CPT, conventional physiotherapy..

Alt text: Paired plot showing Fatigue Severity Scale (FSS) scores at baseline (blue), post-intervention (orange), and follow-up (green) for participants with subacute and chronic stroke in the RAGT+CPT and CPT groups. Individual participant observations are connected by grey lines to indicate repeated measurements. Brackets indicate model-based within-group comparisons with significance annotations.

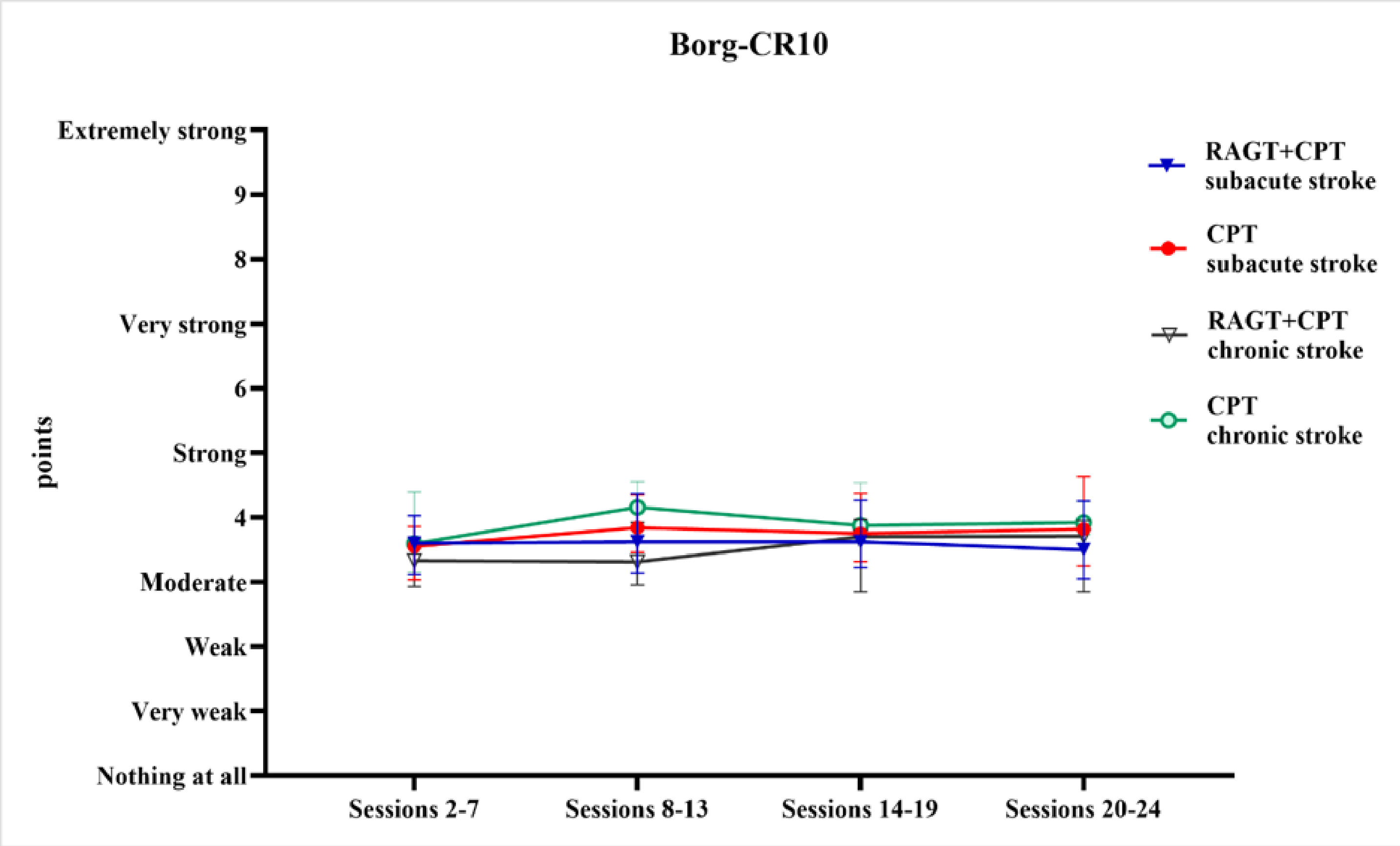

Figure S6. Ratings of perceived exertion during the intervention.

Median Borg CR10 scores across sessions for each of the four study groups. Each line represents a group, with data points indicating the group median at each session. Vertical error bars show the interquartile range (IQR; 25th to 75th percentile), reflecting variability in perceived exertion. The plot illustrates changes in perceived exertion over time and statistically non-significant differences between groups.

Alt text: Line graph of median Borg CR10 ratings of perceived exertion across intervention sessions for the four study groups. Points indicate group medians at each session, connected by lines. Vertical error bars represent the interquartile range (25th to 75th percentile).

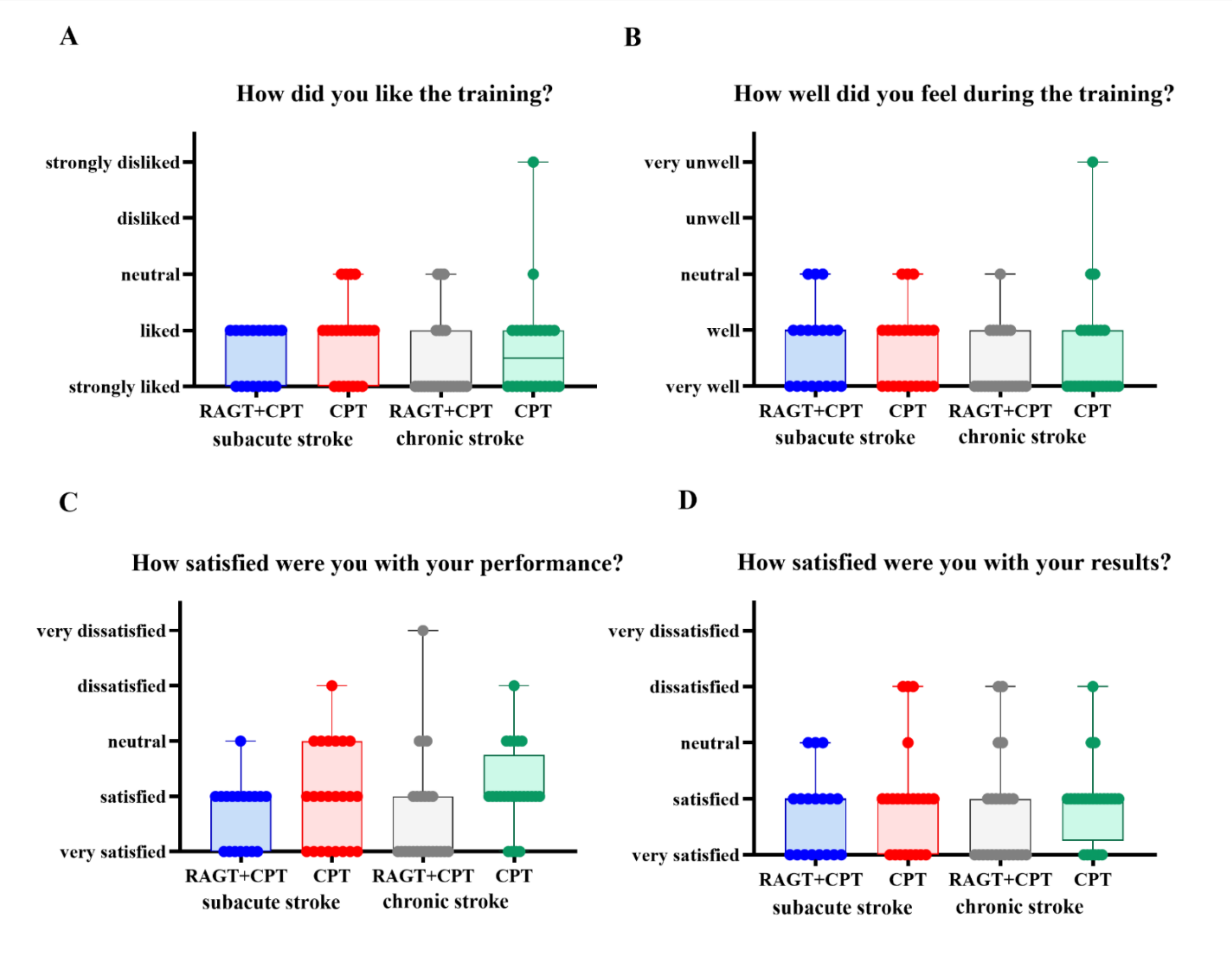

Figure S2. Ratings of acceptability of the intervention

Acceptability of the intervention across four groups, for the acceptability questions: **A** how much participants liked the training; **B** how they felt during the training; **C** how satisfied they were with their performance or **D** with their results. This was assessed using a 5-point Smiley Likert Scale (1 = highest satisfaction; 5 = lowest). For each group, boxplots display the median, interquartile range (IQR), and range, with individual participant responses overlaid as dots.
