# Supplementary material for: Robot-assisted gait training in people with subacute and chronic stroke: a randomized controlled trial
Source: Front Neurol. 2026 Sep 3;17:1898475. doi: 10.3389/fneur.2026.1898475 (PMC13581499; doi:10.3389/fneur.2026.1898475)
Supplement: Supplementary file 3 [file Table_2.DOCX]

**Supplementary Checklist S2.** Template for Intervention Description and Replication (TIDieR) Checklist.

| Item number | Item |
| --- | --- |
|  | **Brief Name** |
| 1 | Robot‑assisted gait training (RAGT) plus conventional physiotherapy (CPT) as compared to time‑matched CPT alone in people with acute and chronic stroke. |
|  | **Why** |
| 2 | The combined intervention was robot‑assisted gait training (RAGT) using the Lexo end‑effector system plus conventional physiotherapy (CPT). The comparator was time‑matched CPT alone. Both interventions target high‑repetition, task‑specific gait practice to optimise motor learning and functional recovery. RAGT provides consistent, intensive stepping with augmented feedback and adjustable body‑weight support to facilitate early practice; CPT delivers overground, context‑specific gait and balance training with graded manual assistance. |
|  | **What** |
| 3 | Materials:  RAGT: Lexo end‑effector robotic gait trainer (Tyromotion GmbH, Graz, Austria) with dynamic body‑weight support (BWS) harness, pelvic guidance, motorised footplates with force sensors, and TyroS software for real‑time augmented feedback (weight bearing, weight shifting, stance/swing phase, initial contact) and parameter control.  CPT: standard inpatient rehabilitation equipment (parallel bars, steps/stairs, balance pads, cones, treadmills/overground walkways, walking aids). |
| 4 | Procedures:  RAGT   - Set‑up: participants secured in a dynamic BWS harness; non‑ambulant participants were lifted into position. The physiotherapist initially assisted paretic knee control and tapered assistance as control improved. - Modes: passive and assistive. Mode selection followed predefined criteria applied at each session. Passive mode was used when the participant could not initiate/sustain stepping or weight shift, had inadequate knee control, or when spasticity, pain or fatigue limited active contribution. Transition to assistive mode required consistent active participation (initiation of swing, controlled stance, reliable weight shift), and adequate knee control. The proportion of passive versus assistive time was progressed within and across sessions. - Adjustable parameters: step length (21–61 cm), cadence 0–100 steps/min (approx. 0–3.6 km/h), pelvis lateral/vertical displacement (0–5 cm), BWS (as required, displayed in software). Starting speed was 1.5 km/h, progressed towards approximately 2.2–2.5 km/h as tolerated. Step length was individualised to achieve a comfortable velocity. Where feasible, BWS was minimised. - Feedback: explicit therapist feedback plus implicit, real‑time visual feedback on distance, duration, step number and length, weight bearing and phase‑specific targets via TyroS. - Progression rules: predefined difficulty bands guided advancement when participants achieved targets at the intended exertion (Borg CR10 score of 3-5 (1)) with knee control and foot clearance: - Easy: BWS up to 100%; passive mode up to 100%; step length 27–39 cm; speed ≤1.4 km/h; distance ≤150 steps; breaks as needed; session time could be reduced by up to 20% if required. - Medium: BWS ≤30%; passive ≤50% and assistive ≤49%; step length 40–50 cm; speed 1.5–2.0 km/h; distance 151–500 steps; session‑time reduction ≤5% if required. - Hard: BWS 0–5%; assistive ≥50%; step length 51–61 cm; speed ≥2.0 km/h; distance ≥500 steps.   CPT covered overground gait training, transfer training, stair negotiation, balance tasks in sitting/standing, and gait‑related strengthening, with progression rules described below.   - Content: individualised, therapist‑supervised gait and balance training integrated within routine inpatient rehabilitation (overground gait training, transfer training, sit‑to‑stand and standing practice, stair negotiation, balance tasks, gait‑related strengthening), with or without parallel bars and/or a walking aid as indicated. - Assistance: the therapist initially assisted paretic knee control and transfers; assistance was tapered as control improved. - Feedback: explicit therapist feedback; implicit feedback via distance walked and task performance. - Progression rules: predefined difficulty bands: - Easy: high assistance for transfers (up to 100%); predominant seated practice (up to 100%); full use of parallel bars (up to 100%); full dependence on an assistive device (up to 100%); step length 25–39 cm; speed ≤1.0 km/h; distance ≤100 steps; session‑time reduction up to 20% if required. - Medium: assistance ≤30% for transfers; seated time ≤30%; use of parallel bars ≤30%; partial independence (assistive device ≤50%, independent walking ≤49%); step length 40–55 cm; speed 1.0–1.5 km/h; distance 101–400 steps; session‑time reduction ≤5% if required. - Hard: minimal assistance (0–5% for transfers); seated time 0–5%; use of parallel bars 0–5%; predominantly independent walking (≥50%); step length 55–80 cm; speed ≥1.5 km/h; distance ≥400 steps. |
|  | **Who Provided** |
| 5 | Sessions were delivered one‑to‑one by licensed physiotherapists.  RAGT: Therapists operating the robot were trained and certified by the manufacturer and the institution’s competency programme.  CPT sessions were provided by experienced physiotherapists who were not involved in outcome assessments. |
|  | **How** |
| 6 | Face‑to‑face, one‑to‑one inpatient sessions. |
|  | **Where** |
| 7 | A dedicated robot room and a dedicated therapy gym within the inpatient rehabilitation facility. |
|  | **When and How Much** |
| 8 | Participants undertook a 4‑week inpatient intervention followed by an 8‑week follow‑up. The weekly schedule was identical in both arms: four sessions of 45 minutes and two sessions of 25 minutes.  In RAGT+CPT, four sessions per week were RAGT (45–50 minutes including set‑up, safety checks, transfers, rest breaks and active practice) and two were CPT (25 minutes).  In CPT‑only, time‑matched CPT was provided (70–75 minutes/session across the weekly schedule) including the same components (set‑up, safety checks, transfers, breaks and active practice).  Breaks were permitted ad libitum. Intensity was monitored using the Borg CR10 every 7–10 minutes to titrate workload and avoid undue fatigue, pain or safety risk. |
|  | **Tailoring** |
| 9 | Both interventions were individualised each session according to clinical status, safety, and perceived exertion (Borg CR10).  For RAGT, therapists adjusted body weight support, mode (passive/assistive proportion), speed, step length and pelvic displacement, following the predefined criteria for mode selection and progression bands.  For CPT, therapists modulated manual assistance, use of parallel bars and assistive devices, task complexity (e.g., surface demands, dual‑task elements), and targeted walking distance and speed. Therapists tapered assistance as knee control and weight shift improved. |
|  | **Modifications** |
| 10 | No protocol modifications occurred after trial initiation. |
|  | **How well** |
| 11 | Planned: Adherence and fidelity were monitored prospectively. Attendance and per‑session duration were recorded for all participants.  RAGT: The Lexo device automatically logged RAGT parameters (BWS, mode, speed, step length, cadence, distance, feedback targets).  CPT content, assistance levels, device use and distances were documented in the electronic therapy record.  Therapists recorded Borg CR10 ratings every 7–10 minutes and any deviations from the progression rules with rationale to ensure consistent decision‑making across participants. |
| 12 | Actual: Participants in RAGT+CPT received a mean of 15 (median 16, range 12–16) RAGT sessions plus eight CPT sessions focused on gait training during the 4‑week inpatient period.  CPT‑only participants received sixteen 45–50‑minute and eight 25‑minute CPT sessions, all targeting gait and balance training.  Reported session durations for both arms included preparation/set‑up, harnessing (RAGT), transfers, safety checks, rest breaks and active practice to ensure parity in allocated therapy time. Intensity was titrated using Borg CR10, and breaks were taken as needed. |

**References**

1.	Harmon EY, Melewski M, Provost D, Sonagere MB. Safety and Benefits of Moderate to High Intensity Aerobic Exercise During the Subacute Phase of Stroke: A Systematic Review and Meta-analysis. Arch Rehabil Res Clin Transl. 2026;8(1):100551.
